# Comparative Mt Genomics of the Tipuloidea (Diptera: Nematocera: Tipulomorpha) and Its Implications for the Phylogeny of the Tipulomorpha

**DOI:** 10.1371/journal.pone.0158167

**Published:** 2016-06-24

**Authors:** Xiao Zhang, Zehui Kang, Meng Mao, Xuankun Li, Stephen L. Cameron, Herman de Jong, Mengqing Wang, Ding Yang

**Affiliations:** 1 Department of Entomology, China Agricultural University, Beijing, 100193, China; 2 Centre for Medical Bioscience, School of Biological Sciences, University of Wollongong, Wollongong, NSW 2522, Australia; 3 Earth, Environmental & Biological Sciences School, Science & Engineering Faculty, Queensland University of Technology, Brisbane, Australia; 4 Naturalis Biodiversity Center Darwinweg 2, 2333 CR, Leiden, The Netherlands; 5 Institute of Plant Protection, Chinese Academy of Agricultural Sciences, Beijing, 100193, China; University of California, Berkeley, UNITED STATES

## Abstract

A traditionally controversial taxon, the Tipulomorpha has been frequently discussed with respect to both its familial composition and relationships with other Nematocera. The interpretation of internal relationships within the Tipuloidea, which include the Tipulidae *sensu stricto*, Cylindrotomidae, Pediciidae and Limoniidae, is also problematic. We sequenced the first complete mitochondrial (mt) genome of *Symplecta hybrida* (Meigen, 1804), which belongs to the subfamily Chioneinae of family Limoniidae, and another five nearly complete mt genomes from the Tipuloidea. We did a comparative analysis of these mt genomics and used them, along with some other representatives of the Nematocera to construct phylogenetic trees. Trees inferred by Bayesian methods strongly support a sister-group relationship between Trichoceridae and Tipuloidea. Tipulomorpha are not supported as the earliest branch of the Diptera. Furthermore, phylogenetic trees indicate that the family Limoniidae is a paraphyletic group.

## Introduction

The animal mitochondrial (mt) genome typically contains 13 protein-coding genes (PCGs), 22 transfer RNA (tRNA) genes, two ribosomal RNA (rRNA) genes and a large non-coding region (also referred to as the control region, or CR) [1]. It is being widely used for understanding the phylogenetic relationships, because it can provide more phylogenetic information than shorter individual nuclear genes and multiple genome-level characteristics, such as modes of control of a replication and transcription, RNA secondary structures. Although there has been some criticism of using mt genomes for phylogenetics as the effects of accelerated substitution rate and compositional heterogeneity may bias topological inferrence, the mt genome is still widely used for understanding the phylogenetic relationships in many insect groups, such as the Paraneoptera, Megaloptera, Coleoptera, and Orthoptera [2–5]. The number of sequenced mt genomes has rapidly increased over the years, especially in the Diptera. By June 2015, there had been 118 complete and nearly complete Diptera mt genome sequences which were available in GenBank, including 45 nematoceran species representing 14 families. About half of these genomes were from species which belong to the Culicidae; the other half, which were mostly sequenced by two studies [6–7], represented the families Anisopodidae, Cecidomyiidae, Ceratopogonidae, Chironomidae, Dixidae, Keroplatidae, Pachyneuridae, Psychodidae, Ptychopteridae, Sciaridae, Tanyderidae, Tipulidae and Trichoceridae. A summary of available mt genome sequences from the Nematocera is given in Table 1. Among these sequences, however, only two complete and one nearly complete mt genomes representing the Tipulomorpha were available.

**Table 1:** List of Taxon Included in This Study.

Order	Family	Species	Length (bp)	Accession No.	Reference
Diptera	Culicidae	*Anopheles quadrimaculatus* Say	15455	NC_000875	Mitchell *et al*. (1993) [8]
		*Anopheles gambiae* Giles	15363	NC_002084	Beard *et al*. (1993) [9]
		*Anopheles darling* Root*	15386	NC_014275	Moreno *et al*. (2010) [10]
		*Anopheles culicifacies* Giles	15330	NC_027502	Hua, YQ. *et al*. (2015) [11]
		*Anopheles cruzii* Dyar & Knab	15449	NC_024740	Marinotti, O. *et al*.
		*Anopheles deaneorum* Rosa-Freitas	15424	NC_020663	Krzywinski *et al*. (2011) [12]
		*Anopheles albitarsis* Lynch-Arribálzaga*	15413	NC_020662	Krzywinski *et al*. (2011) [12]
		*Anopheles oryzalimnetes* Wilkerson & Motoki	15422	HQ335345	Krzywinski *et al*. (2011) [12]
		*Anopheles janconnae* Wilkerson & Sallum	15425	HQ335348	Krzywinski *et al*. (2011) [12]
		*Anopheles farauti* Laveran	15412	NC_020770	Logue *et al*. (2013) [13]
		*Anopheles hinesorum* Schmidt	15336	NC_020769	Logue *et al*. (2013) [13]
		*Anopheles cracens* Sallum & Peyton	15412	NC_020768	Logue *et al*. (2013) [13]
		*Anopheles dirus* Peyton & Harrison	15404	JX219731	Logue *et al*. (2013) [13]
		*Anopheles punctulatus* Donitz	>15412	JX219744	Logue *et al*. (2013) [13]
		*Anopheles koliensis* Owen	>15412	JX219743	Logue *et al*. (2013) [13]
		*Aedes albopictus* (Skuse)	16665	NC_006817	Ho *et al*. unpublished
		*Aedes aegypti* (Linnaeus)	16655	NC_010241	Behura *et al*. (2011) [14]
		*Aedes notoscriptus* (Skuse)	15846	NC_025473	Hardy, C.M. *et al*. (2014) [15]
		*Aedes vigilax* (Skuse)	15877	KP995260	Hardy, C.M. *et al*. (2015) [16]
		*Culex quinquefasciatus* Say*	15587	NC_014574	Behura *et al*. (2011) [14]
		*Culex pipiens* Linnaeus	14856	NC_015079	Atyame *et al*. (2011) [17]
		*Ochlerotatus vigilax* (Skuse)	15877	NC_027494	Hardy, C.M. *et al*. (2015) [15]
	Ceratopogonidae	*Culicoides arakawae* (Arakawa)*	18135	NC_009809	Matsumoto *et al*. (2009) [18]
	Sciaridae	*Bradysia amoena* (Winnertz)*	>14049	GQ387651	Beckenbach & Joy (2009) [6]
	Cecidomyiidae	*Mayetiola destructor* (Say)*	14759	NC_013066	Beckenbach & Joy (2009) [6]
		*Rhopalomyia pomum* Gagne*	14503	NC_013063	Beckenbach & Joy (2009) [6]
	Trichoceridae	*Trichocera bimacula* Walker*	16140	NC_016169	Beckenbach (2012) [7]
		*Paracladura trichoptera* (Osten Sacken)*	16143	NC_016173	Beckenbach (2012) [7]
	Anisopodidae	*Sylvicola fenestralis* (Scopoli)*	16234	NC_016176	Beckenbach (2012) [7]
	Tipulidae	*Tipula abdominalis* (Say)*	>14566	JN_861743	Beckenbach (2012) [7]
	Ptychopteridae	*Ptychoptera* sp.*	15214	NC_016201	Beckenbach (2012) [7]
		*Bittacomorphella fenderiana* Alexander*	>15609	JN_861745	Beckenbach (2012) [7]
	Tanyderidae	*Protoplasa fitchii* Osten Sacken*	16154	NC_016202	Beckenbach (2012) [7]
	Pachyneuridae	*Cramptonomyia spenceri* Alexander*	16274	NC_016203	Beckenbach (2012) [7]
	Keroplatidae	*Arachnocampa flava* Harrison*	16923	NC_016204	Beckenbach (2012) [7]
	Chironomidae	*Chironomus tepperi* Skuse*	15652	NC_016167	Beckenbach (2012) [7]
		*Parochlus steinenii* Gercke	16803	KT003702	Shin, SC. & Kim, SH.
	Dixidae	*Dixella* sp.*	15574	KM245574	Kang *et al*. (2014) [19]
	Psychodidae	*Nyssomyia umbratilis* (Ward & Fraiha)*	15757	NC_026898	Kocher, A *et al*. (2015) [20]
	Limoniidae	*Symplecta hybrida* (Meigen)*	15811	KT970064	Present study
		*Rhipidia chenwenyoungi* Zhang, Li & Yang *	>14647	KT970063	Present study
		*Paradelphomyia* sp.*	>14636	KT970061	Present study
	Cylindrotomidae	*Cylindrotoma* sp.*	>15372	KT970060	Present study
	Pediciidae	*Pedicia* sp.*	>14829	KT970062	Present study
	Tipulidae	*Tipula cockerelliana* Alexander*	>14541	KT970065	Present study
Brachycera (suborder)	Tabanidae	*Cydistomyia duplonotata* (Ricardo)*	16247	NC_008756	Cameron, *et al*. 2007 [21]
	Nemestrinidae	*Trichophthalma punctata* (Macquart)*	16396	NC_008755	Cameron, *et al*. 2007 [21]
	Syrphidae	*Simosyrphus grandicornis* (Macquart)*	16141	NC_008754	Cameron, *et al*. 2007 [21]
	Muscidae	*Haematobia irritans* (Linnaeus)*	16078	NC_007102	Lessinger, *et al*. unpublished
Mecoptera	Boreidae	*Boreus elegans* Carpenter*	16803	NC_015119	Beckenbach 2011[22]
	Bittacidae	*Bittacus pilicornis* Westwood*	15842	NC_015118	Beckenbach 2011[22]
Siphonaptera	Ceratophyllidae	*Jellisonia amadoi* Ponce-Ulloa*	17031	NC_022710.1	Cameron, 2013 [23]

* Species used for phylogenetic analysis in this study.

The Tipulomorpha is a controversial group with both its familial composition and relationships to other Nematocera disputed by different workers [24]. The Tipulomorpha has been defined to include both the Tipulidae *sensu lato* (the Tipuloidea or crane flies) and the Trichoceridae (winter crane flies) [25–29] or just the Tipulidae *sensu lato* [30]. A taxonomically diverse group, the Tipulidae *sensu lato* (sometimes defined to include the families Cylindrotomidae, Limoniidae, Pediciidae, and Tipulidae *sensu stricto*), have been recorded worldwide, and have 15412 described species [31]. Adults of this group are easily recognized by their slender bodies and extremely long legs in combination with two well-developed anal veins on the wings. They usually live in moist, temperate environments, and are often found in herbaceous vegetation near streams and lakes in the forested areas. The larvae of crane flies live in various environments, including freshwater, marshes, moist soil and decaying wood [32]. The Trichoceridae are superficially similar, small slender flies with long legs. They are different from the Tipulidae *sensu lato* by the existence of three ocelli and a relatively short A_2_ vein; some larval characters are also not found in the tipuloids, such as the conical labrum and the divided mandible [33–34].

The phylogenetic relationships of Tipulomorpha within the Nematocera are also controversial. The idea that the Tipulomorpha includes both the Tipulidae *sensu lato* and the Trichoceridae was advocated by Hennig, who also suggested a sister-group relationship between Tipulomorpha and all remaining Diptera [25–27]. This relationship was also suggested by several other studies [35–37]. The sister-group relationship of Tipulidae *sensu lato* and Trichoceridae was mainly supported by morphologic characters of the adults [38]. Wood and Borkent restricted the Tipulomorpha to the Tipuloidea excluding Trichoceridae, which they also considered the sister-group to all other Diptera. According to Wood & Borkent, the Trichoceridae belonged to the Psychodomorpha based on larval morphology [30]. This position of Trichoceridae, as proposed by Wood and Borkent, was subsequently accepted by Griffiths, but he suggested that the tipuloid families should be moved from the earliest branch of the dipteran phylogenetic tree and nested within Psychodomorpha [28]. This subordinate position of the tipuloids was also suggested by Oosterbroek and Courtney [29]. In the Bayesian consensus analysis of morphology by Lambkin *et al*. [39] Trichoceridae was sister to a clade composed of Psychodidae + Bibionomorpha [39]. Yeates *et al*. [24] suggested that Tipulomorpha was paraphyletic, with Trichoceridae nested within Psychodomorpha in their supertree analysis. They recovered a restricted Tipulomorpha (equal to Tipuloidea alone) as the sister-group to the Brachycera [24]. In the first molecular phylogenetic analysis of deep-level dipteran relationships, using the 28S rRNA gene, Tipulomorpha was also paraphyletic [40], while the more recent, multigene analysis by Wiegmann *et al*. [41] suggested that Tipulomorpha included Trichoceridae. Recent transcriptome-based phylogenetic trees indicated that the Tipulomorpha (represented by Tipulidae alone as Trichoceridae has not been subject to RNAseq analysis yet) represented neither the earliest branching dipteran infraorder, nor the most derived branch of the lower Diptera (= Nematocera) [42–43].

Since there is only one nearly complete mt genome sequence of Tipuloidea (*Tipula abdominalis*, JN_861743) available in GenBank (as of June 2015) [6], we sequenced and described the first complete and another five nearly complete mt genomes from Tipuloidea (Table 1), representing its four families (Cylindrotomidae, Limoniidae, Pediciidae and Tipulidae *sensu stricto*). We annotated these genomes and did a comparative analysis of these mt genomics. Using these new sequences, along with published representatives of the Nematocera, we constructed phylogenetic trees of the Tipulomorpha. The implications of the phylogenetic relationship between Trichoceridae and Tipuloidea, and the position of the Tipulomorpha in the lower Diptera were given in this paper.

## Materials and Methods

### Ethics statement

No specific permits were required for the specimens collected for this study. The specimens were collected by net. The specimens were common in China and the field studies did not involve endangered or protected species. The species were not included in the “List of Protected Animals in China”.

### Specimen collection and preparing

All specimens used for DNA extraction were collected from China. The details of the collection information were listed in S1 Table. Specimens were initially preserved in 95% EtOH in the field, and then transferred to -20°C for the long-term storage at China Agricultural University (CAU). Specimens were identified by Zehui Kang (CAU).

### DNA extraction, PCR and Sequencing

Thoracic muscle tissues were removed for extraction of whole genomic DNA using the TIANamp Genomic DNA Kit (TIANGEN). The mt genomes of six species were amplified using NEB Long Taq DNA polymerase (New England Biolabs, Ipswich, MA). First, fragments of 500–1500 bp were amplified using standard primers conserved across insects [44]. Additional sequences were obtained using taxon-specific primers designed based on these preliminary sequence. The details of primers information are listed in S2 Table. PCR amplification conditions are as follows: a hot-start denaturation step at 95°C for 30sec; 40 cycles of denaturation at 95°C for 10sec; annealing at 40–55°C for 50sec; extension at 65°C for 1kb/min; final elongation step at 65°C for 10min. The quality of PCR products was evaluated by electrophoresis in a 1% agarose gel stained with Gold View nucleic acid stain. Purified PCR amplicons were sequenced in both directions using the BigDye Terminator Sequencing Kit ver. 3.1(Applied Bio Systems) and ABI 3730XL Genetic Analyzer (PE Applied Biosystems, San Francisco, CA, USA) using both amplification and internal primers designed via primer walking.

### Bioinformatic and Phylogenetic analysis

Sequences were assembled manually. First, sequences were identified and aligned into contigs using BioEdit version 7.0.5.3 [45]. After fully assembling each mt genome, we identified the protein-coding genes as open reading frames and by alignment with homologous sequences annotated in the mt genomes of 45 published nematoceran species. The tRNA genes were identified using tRNAscan-SE [46], and analyzed with a COVE score cutoff of 1 for identifying all possible tRNA genes. Those tRNA genes (the *tRNA*^*Ser(AGN)*^ of six sequenced species) that could not be identified using tRNAscan and the rRNA genes were identified by alignment with homologous sequences from the 45 published nematoceran species. MEGA 5.0 [47] was used to analyze the nucleotide substitution rates, base composition and codon usage. Nucleotide compositional skew was calculated using the formulae: AT-skew = (A-T)/ (A+T); GC-skew = (G-C)/ (C+G) [48].

A phylogenetic analysis was conducted using a total of 29 species of Diptera as an ingroup and three outgroup species from Diptera’s close relatives, *Bittacus pilicornis*, Westwood (NC_015118) and *Boreus elegans*, Carpenter (NC_015119) of Mecoptera and *Jellisonia amadoi*, Ponce-Ulloa of Siphonaptera [22–23]. Details of the species used for phylogenetic analysis in this study are listed in Table 1.

Because *tRNA*^*Ile*^, *tRNA*^*Gln*^ and *tRNA*^*Met*^ were not sequenced for the 5 species whose incomplete mt genomes were obtained, the phylogenetic analyses only include the remaining 19 tRNAs, 13 PCGs, lrRNA, and a portion of srRNA (the alignment was trimmed to exclude the missing regions of srRNA). Each gene was aligned in MEGA 5.0 [47] based on the annotation procedures proposed by Cameron [49]. Individual genes were concatenated into a single data matrix using SequenceMatrix v1.7.8 [50]. Two datasets were assembled for phylogenetic analyses: the first dataset consisted of the first and second codon positions of the 13 PCGs (PCG12), two rRNAs and 19 tRNAs (PCG12RNA); the second dataset consisted of the first and second codon positions of five PCGs (*CO1*, *CO2*, *CO3*, *CYTB* and *ATP6*), which was excluded the remaining eigth difficult aligned genes(5PCG12RNA) [51], two rRNAs and 19 tRNAs (5PCG12RNA). The two aligned datasets were 9815 and 6067 bp long for the PCG12RNA and the 5PCG12RNA matrices respectively. We used PartitionFinder v1.1.1 [52] to select the best-fit partitioning scheme and the substitution models for each partition. The best-fit partitioning scheme for constructing phylogenetic tree is listed in S3 Table.

Bayesian inference (BI) was used for phylogenetic analyses. BI was conducted using MrBayes 3.2.2 [53] for 2–4 million generations. We considered that the stationarity was reached when the average standard deviation of split frequencies between runs was below 0.01, which was tested using AWTY [54].

## Results and Discussion

### General Features of the Genomes

**Fig 1:**
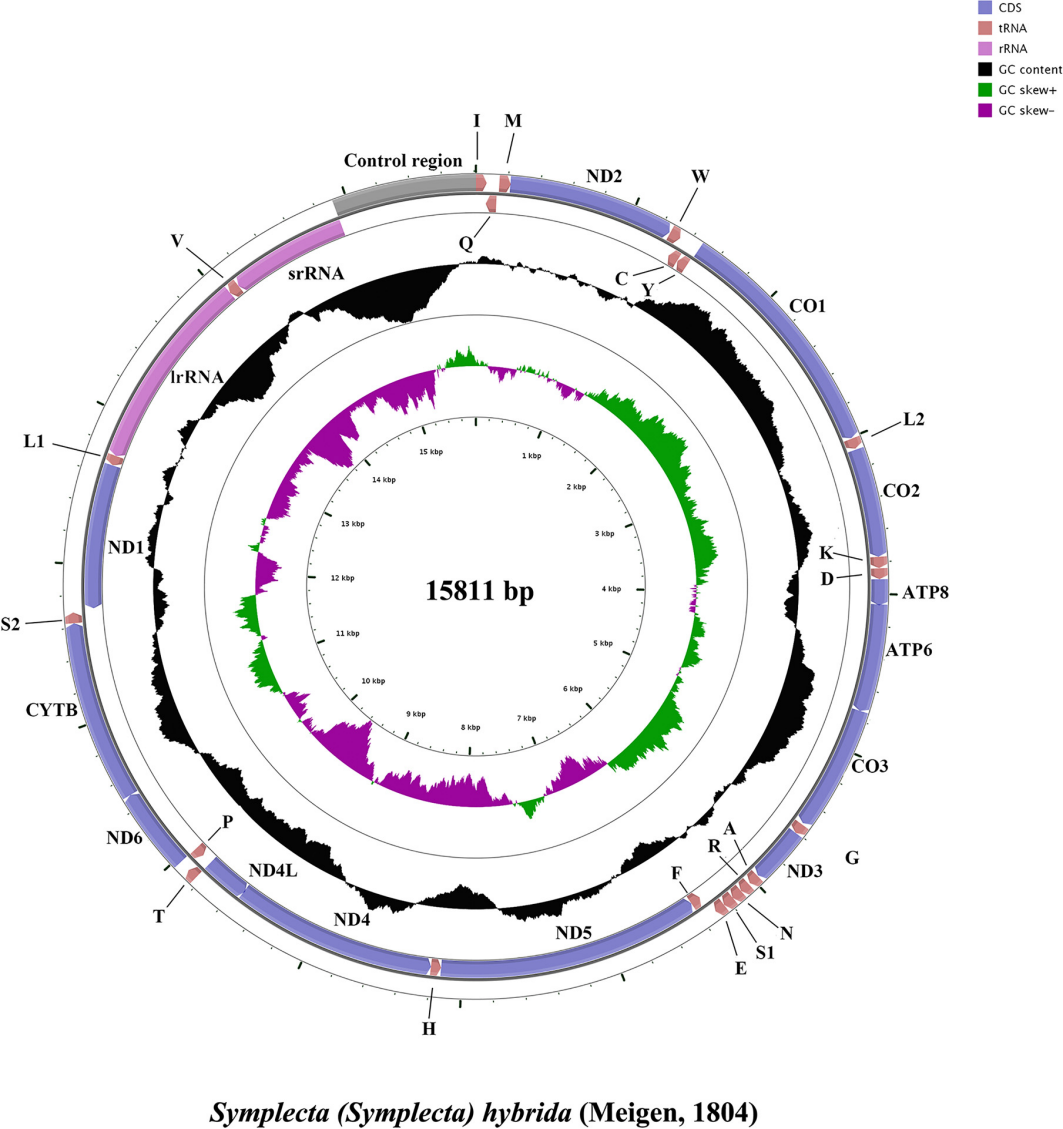
Mitochondrial map of *Symplecta hybrida*. Circular maps were created using CGView [55]. The outermost circle shows the gene arrangement and comparison, and the arrows indicated the orientation of gene transcription. The tRNAs are abbreviated according to the IUPACIUB single-letter amino acid codes (S1: AGN; S2: UCN; L1: CUN; L2: UUR). The second circle (a black sliding circle) shows the GC content, as the deviation from the average GC content of the entire sequence. The third circle indicated the GC-skew, as the deviation from the average GC-skew of the entire sequence. The inner cycle indicated the size and the location of the genes.

Six mt genomes of Tipuloidea were sequenced: *Cylindrotoma* sp. (15372bp), *Paradelphomyia* sp. (14639bp), *Pedicia* sp. (14605bp), *Rhipidia chenwenyoungi* (13809bp), *Symplecta hybrida* (15,811bp) and *Tipula cockerelliana* (14453bp) (GenBank accession number: KT970060–KT970065). The mt genome of *S*. *hybrida* was complete and the remaining five were nearly complete. The newly sequenced complete mt genome fall within the middle of the size range previously reported for mt genomes from the Nematocera, which ranges from 15,214bp in *Ptychoptera* (Ptychopteridae) to about 18,600bp in *Bittacomorphella* (Ptychopteridae) [6]. All mt genomes are typical of insect mt genomes in gene content: 13 protein-coding genes, 22 tRNA genes and two rRNA genes. Gene order is also identical to that of the ancestral insect mt genome, with 23 genes encoded on the majority strand (J-strand), and the remaining 14 genes encoded on the minority strand (N-strand) (Fig 1).

Three conserved regions were found in overlapping regions of genes of each sequenced Tipuloidea: AARYYTTA (*tRNA*^*Trp*^-*tRNA*^*Cys*^), ATGATTA (*ATP8*-*ATP6*) and TTAACAT (*ND4*-*ND4L*). These conserved regions are also shared with some other Diptera [56]. Furthermore, there were also two non-coding intergenic regions conserved in dipteran insects, which have been shown to be binding sites for a bidirectional transcription termination factor (DmTTF) [6]. The first one is located between *tRNA*^*Glu*^ and *tRNA*^*Phe*^ and ranges from 19 bp to 32 bp in length. This intergenic region is absent in other insect orders and not completely conserved in Diptera [6]. In Diptera, this intergenic region is present in all Brachycera and some Nematocera, being absent in Culicidae [6]. The second intergenic region is found between *tRNA*^*Ser(UCN)*^ and *ND1* and ranges from 16bp to 38bp in length (S4 Table). It is highly conserved across insects and similar sequences for this region are present in other orders, such as Mecoptera [6, 57].

The control region (CR) is the longest intergenic region in the mt genome. Only one complete control region from *S*. *hybrida* was sequenced in this study. It is 897bp in length and located in the conserved position between *srRNA* and *tRNA*^*Ile*^ [56]. It is a medium-sized CR in the Nematocera, where the length of the control region ranges from 369bp in *Ptychoptera* to about 3.7kb in *Bittacomorphella* [6]. We did not find any conserved features identified in other insect CRs, such as poly-T stretch, (TA)_n_ like stretch or stem-loop structure at the 3’-end of the control region [56, 58]. However, we identified three tandem repeat copies of a sequence within the CR with a total length of 174bp. The second and third repeat units are identical in sequence while the first is much shorter at only 46 bp. Large tandem repeats in the control region are common in the Nematocera, for example, Beckenbach detected such repeats in five nematoceran species (*Sylvicola fenestralis*; *Cramptonomyia spenceri*; *Protoplasa fitchii*; *Arachnocampa flava*; *Bittacomorphella fenderiana*)[6].

### Base composition

As with other insects, the nucleotide composition of the tipuloid mt genomes are biased towards A and T [6, 56]. In general, the AT content of these mt genomes are intermediate for nematocerans, in which AT content ranges from about 73% in the *Trichocera* (Trichoceridae) to about 83% in Cecidomyiidae [6]. For protein-coding genes, the AT content of N strand genes (average content: 76.8%) is higher than that of the J strand genes (average content: 72.9%). The AT content of PCG third codon positions is much higher than that of the first and second codon positions. For RNA genes, the average AT content (81.5%) of the lrRNA is slightly higher than that of the srRNA (75.9%). Each of the six tipuloid mt genomes overall has a weakly positive AT-skew and a negative GC-skew on the J-strand, while for PCGs T content is higher than A content. Of each codon position in the PCGs, AT-bias is strongest at the second codon position. Statistics also indicated that the AT-bias is stronger in RNA-encoding genes than in PCGs (Table 2).

**Table 2:** mitochondrial nucleotide composition in six tipuloid flies.

Region		*Cylindrotoma*	*Paradelphomyia*	*Pedicia*	*Rhipidia*	*Symplecta*	*Tipula*
**PCGs(J)**	A+T%	**72.7**	**75**	**70.2**	**74.9**	**74**	**70.6**
	G+C%	**27.2**	**25.1**	**29.9**	**25.1**	**26**	**29.4**
	AT-skew	**-0.09**	**-0.11**	**-0.06**	**-0.13**	**-0.13**	**-0.11**
	GC-skew	**-0.14**	**-0.08**	**-0.15**	**-0.08**	**-0.06**	**-0.18**
**1stcondon position(J)**	A+T%	**66.9**	**68.1**	**62**	**67.9**	**64.8**	**63.6**
	G+C%	**33.5**	**31.6**	**38**	**32.2**	**35.2**	**36.7**
	AT-skew	**-0.02**	**0.03**	**0.03**	**-0.03**	**-0.11**	**-0.01**
	GC-skew	**0.18**	**0.17**	**0.11**	**0.2**	**0.16**	**0.09**
**2ndcondon position(J)**	A+T%	**64.3**	**66.3**	**64.3**	**65.9**	**67.8**	**64.5**
	G+C%	**35.4**	**33.6**	**35.5**	**34.4**	**32.2**	**35.1**
	AT-skew	**-0.37**	**-0.36**	**-0.37**	**-0.37**	**-0.3**	**-0.36**
	GC-skew	**-0.27**	**-0.24**	**-0.24**	**-0.25**	**-0.25**	**-0.26**
**3rdcondon position(J)**	A+T%	**87.5**	**90.2**	**83.8**	**91.8**	**89.7**	**83.7**
	G+C%	**12.5**	**9.8**	**16.1**	**8.7**	**10.3**	**16.7**
	AT-skew	**0.04**	**-0.04**	**0.12**	**-0.05**	**-0.03**	**0**
	GC-skew	**-0.6**	**-0.3**	**-0.55**	**-0.49**	**-0.2**	**-0.59**
**PCGs(N)**	A+T%	**77.5**	**78.3**	**74.2**	**78.7**	**78.1**	**74.5**
	G+C%	**22.5**	**21.7**	**25.8**	**21.2**	**21.9**	**25.5**
	AT-skew	**-0.24**	**-0.22**	**-0.32**	**-0.17**	**-0.2**	**-0.22**
	GC-skew	**0.26**	**0.28**	**0.33**	**0.25**	**0.25**	**0.34**
**1stcondon position(N)**	A+T%	**74**	**74.1**	**70.6**	**74.3**	**72.8**	**77.5**
	G+C%	**26.1**	**25.8**	**29.7**	**25.4**	**27.2**	**22.8**
	AT-skew	**-0.19**	**-0.16**	**-0.19**	**-0.1**	**-0.15**	**-0.16**
	GC-skew	**0.47**	**0.43**	**0.45**	**0.45**	**0.47**	**0.48**
**2ndcondon position(N)**	A+T%	**68.7**	**69.4**	**68**	**69.3**	**68.3**	**69.2**
	G+C%	**31.6**	**30.4**	**32.4**	**31**	**31.7**	**31.2**
	AT-skew	**-0.43**	**-0.44**	**-0.44**	**-0.41**	**-0.43**	**-0.3**
	GC-skew	**0**	**0.05**	**0.01**	**0.01**	**-0.01**	**0.26**
**3rdcondon position(N)**	A+T%	**90.3**	**91.6**	**85.1**	**92.8**	**93.4**	**77.1**
	G+C%	**9.7**	**8.9**	**15.2**	**7.4**	**6.6**	**22.6**
	AT-skew	**-0.15**	**-0.09**	**-0.34**	**-0.03**	**-0.07**	**-0.22**
	GC-skew	**0.59**	**0.62**	**0.76**	**0.54**	**0.59**	**0.3**
**tRNA genes**	A+T%	**78.1**	**80.9**	**75**	**80.3**	**75.9**	**75.8**
	G+C%	**21.9**	**19.1**	**25**	**19.7**	**24.1**	**24.1**
	AT-skew	**0**	**0.02**	**0**	**-0.01**	**0**	**0.02**
	GC-skew	**0.12**	**0.14**	**0.09**	**0.16**	**0.13**	**0.09**
**lrRNA**	A+T%	**82**	**83**	**80.1**	**82.5**	**81.5**	**80.1**
	G+C%	**18**	**17**	**19.9**	**17.5**	**18.5**	**19.9**
	AT-skew	**-0.04**	**-0.1**	**-0.09**	**-0.01**	**-0.05**	**-0.03**
	GC-skew	**0.29**	**0.31**	**0.3**	**0.28**	**0.29**	**0.36**
**srRNA**	A+T%	**79.3**	**78.9**	**74.6**	**78.7**	**79.2**	**75.7**
	G+C%	**20.7**	**21.1**	**25.5**	**21.3**	**20.8**	**24.3**
	AT-skew	**-0.03**	**-0.03**	**0**	**-0.01**	**-0.01**	**0.02**
	GC-skew	**0.26**	**0.27**	**0.26**	**0.23**	**0.25**	**0.25**
**Whole mitgenome**	A+T%	**76.7**	**77.5**	**73.1**	**77.5**	**77.1**	**73.3**
	G+C%	**23.3**	**22.4**	**26.9**	**22.4**	**22.9**	**26.6**
	AT-skew	**0.03**	**0.02**	**0.08**	**0**	**0.01**	**0.02**
	GC-skew	**-0.18**	**-0.16**	**-0.21**	**-0.16**	**-0.14**	**-0.23**

Note: The AT-bias and GC-bias of PCGs were calculated by the formulae: AT-skew = (A-T)/(A+T), GC-skew = (G-C)/(C+G).

### Codon usage

Codon usage for the six tipuloid species is shown in S5 Table. The AT rich codons TTA (Leu), ATT (Ile), TTT (Phe), ATA (Met), AAT (Asn) and TAT(Tyr) are the most frequently used codons.

**Fig 2:**
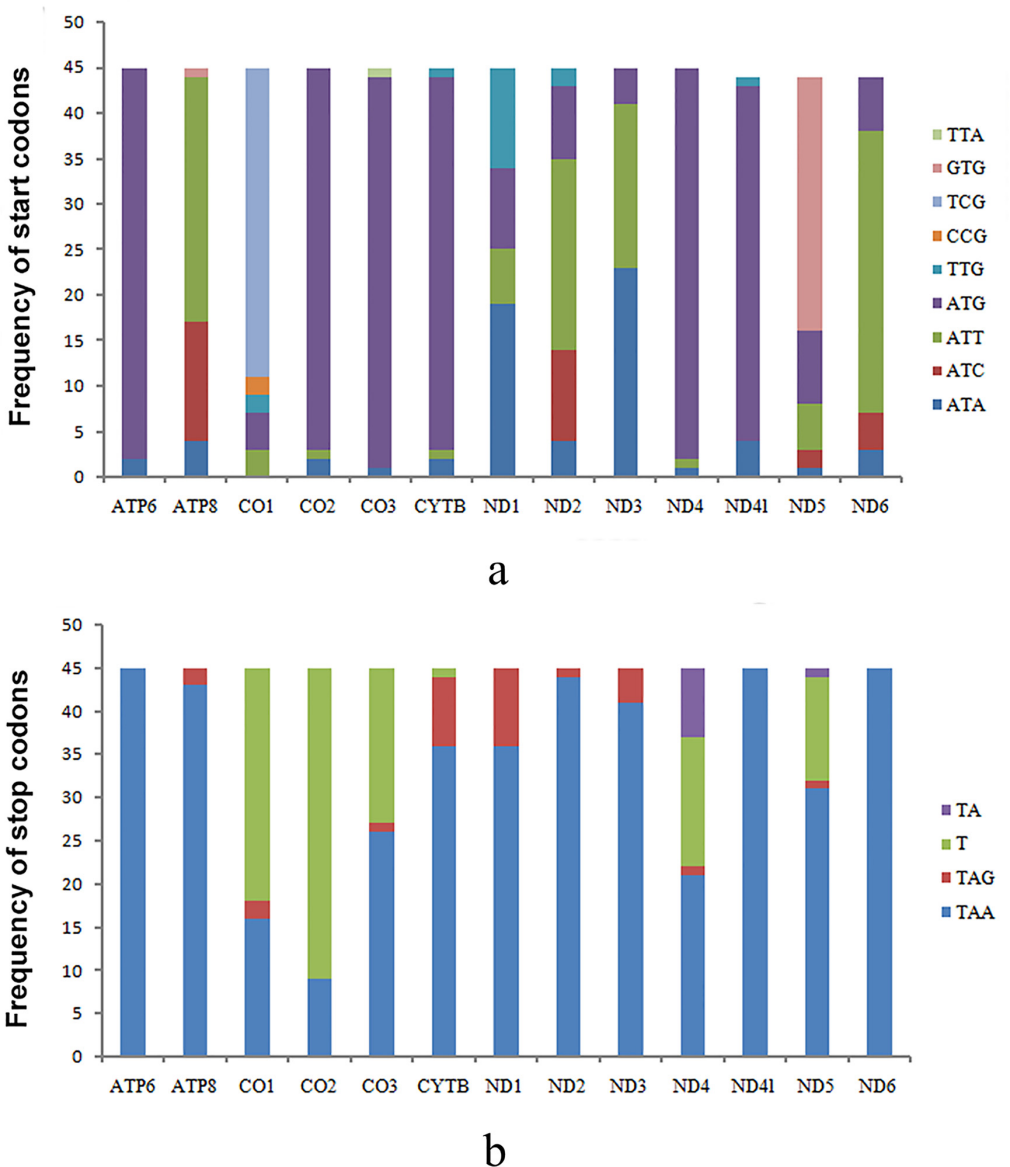
Start and stop codons usage in nematoceran mt genomes. a: Start codons usage of PCGs in Nematocera; b: Stop codons usage of PCGs in Nematocera.

Among all sequenced nematoceran flies, the most commonly used start codons are the canonical start codons ATN (Met/Ile), found in every PCG. Among them, ATG (Met) and ATT (Ile) are the mostly common used start codons. ATG (Met) is used in *ATP6*, *CO2*, *CO3*, *CYTB*, *ND4* and *ND4L* for almost all nematoceran flies. This pattern is also observed in cyclorrhaphan flies [56]. ATT (Met) is found in 10 of the 13 PCGs (*ATP6*, *ATP8*, *CO1*, *CO3*, *ND2*, *ND4L*, and *ND6*) for almost all nematoceran flies, especially used for *ND2*, *ATP8*, *ND3*, and *ND6*. However, another two conventional start codons ATA (Met) and ATC (Ile) are found in a minority of the nematoceran species. GTG (Val), TCG (Ser^UCN^) and TTG (Leu^UUR^) are used for *ND5*, *CO1* and *ND1* respectively in most species. CCG (Pro) is identified as the start codon for *CO1* in *C*. *arakawae* (Ceratopogonidae) and *Dixella* sp. (Dixidae). TTA (Leu^UUR^) is the start codon of *CO3* in *R*. *pomum* (Cecidomyiidae) (Fig 2, S6 Table).

Similar to most other Diptera, the most commonly used stop codon in tipuloids is TAA, which was found in 7 of the 13 PCGs (*ATP6*, *ATP8*, *CO1*, *CO3*, *ND2*, *ND4L*, and *ND6*) for almost all tipuloids. The stop codon TAG is used in almost all the *ND1* and also can be found in *ND3* and *CYTB*. All the *CO2* genes in tipuloids use the partial stop codon T, and the two remaining PCGs (*ND5* and *ND4*) of tipuloids’ mt genomes usually have the partial stop codon T or TA. In all sequenced nematoceran flies, TAA is also the most commonly used stop codon that found in every PCG, especially in *ATP6*, *ND4l*, *ND6*, *ATP8*, *ND1*, *ND2* and *ND3*. All the *ATP6*, *ND4l*, *ND6* of nematoceran flies used TAA as stop codon, and TAG were found in a minority of *ATP8*, *ND1*, *ND2* and *ND3*. Two partial stop codons, T or TA, are found in *CO1*, *CO2*, *CO3*, *ND4* and *ND5*, especially in *CO1* and *CO2*. In *ND4* and *ND5*, there are two kind of partial stop codons (T or TA). (Fig 2, S6 Table).

### Transfer and ribosomal RNAs

**Fig 3:**
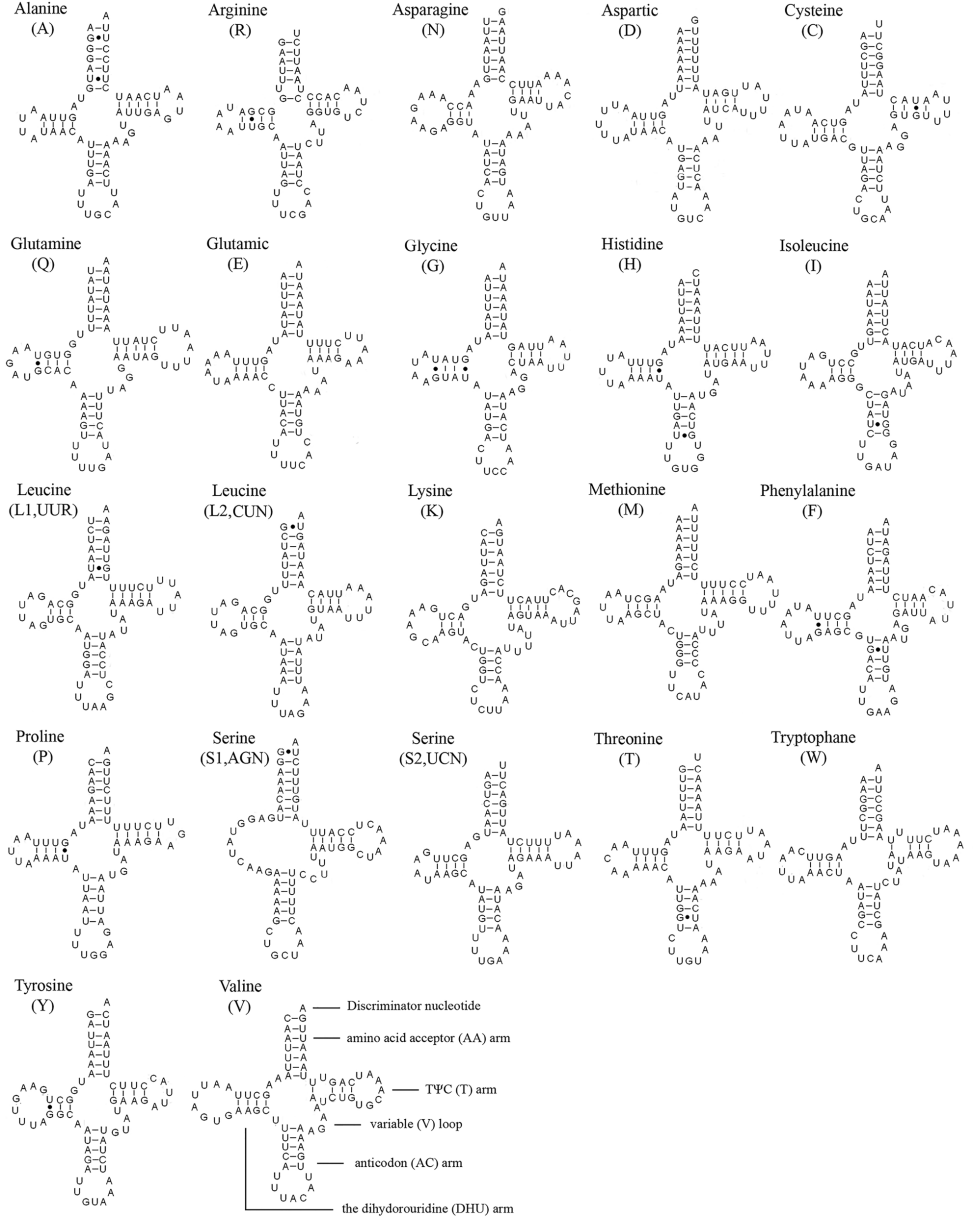
Inferred secondary structures of tRNAs found in the mt genome of *Symplecta hybrida*. All tRNAs can be folded into a clover-leaf secondary structure. All tRNAs are labelled with the abbreviations of their corresponding amino acids. The short line indicated the inferred Watson-Crick bonds, and the dark dots indicated GU bonds.

All 22 tRNA genes in *S*. *hybrida* and 19 of the 22 tRNA genes in the remaining five tipuloids were identified. The length of mt tRNAs ranges from 64 bp to 72 bp. Most tRNA genes can be folded into a typical clover-leaf secondary structure (Fig 3), whereas *tRNA*^*Ser(AGN)*^ is an exception for lacking a DHU arm [59]. Some mispairings (U–U and G–U) are found in tRNAs. For example, four mismatched base U–U pairs and 17G–U pairs are found in tRNA secondary structures in *S*. *hybrida*, while no other types of mispairings are found.

**Fig 4:**
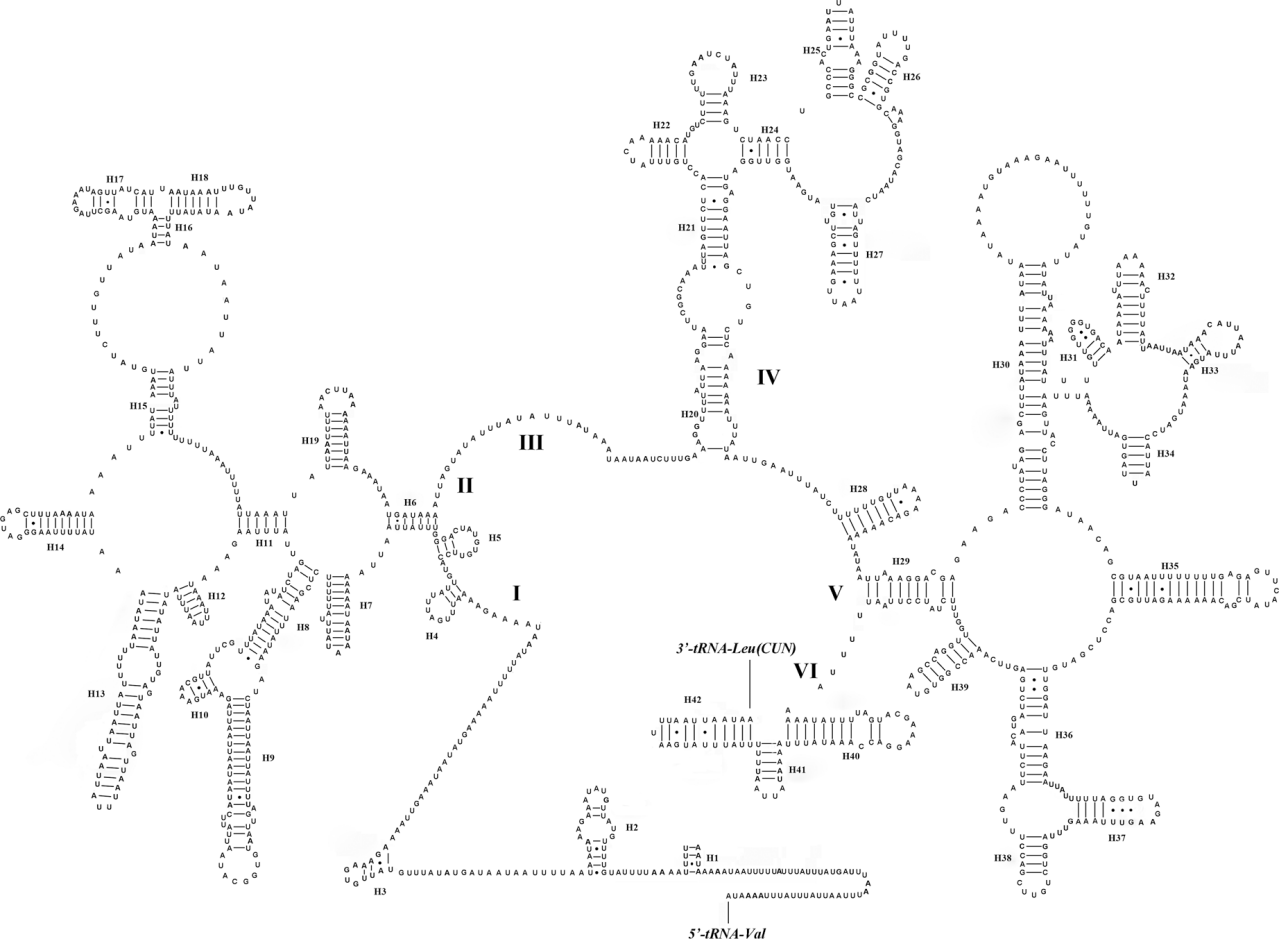
Inferred secondary structure of the *lrRNA* gene in *Symplecta hybrida*. The short line indicated the inferred Watson-Crick bonds, and the dark dots indicated GU bonds.

**Fig 5:**
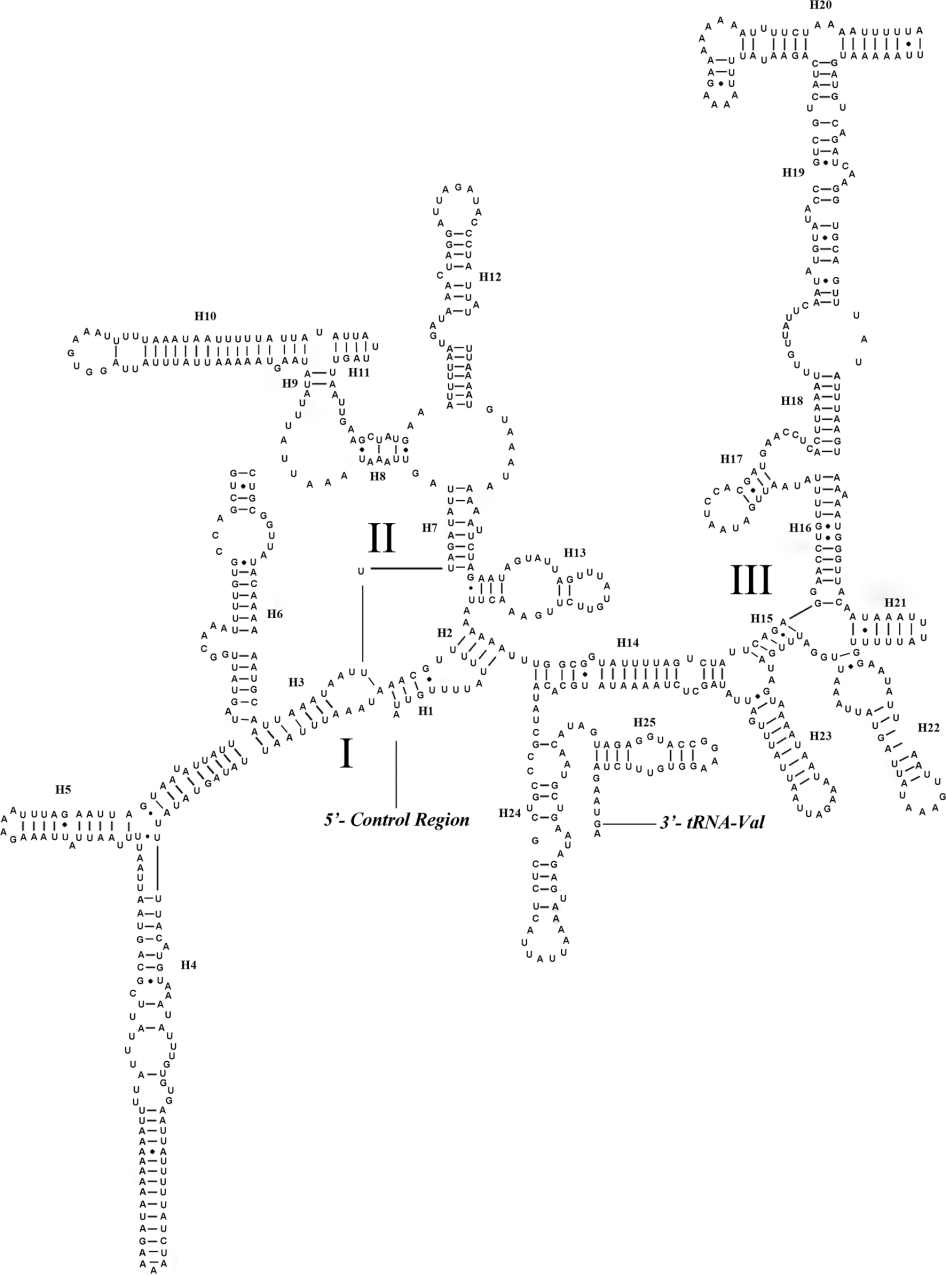
Predicted secondary structure of the *srRNA* gene in *Symplecta hybrida*. The conserved domain structures are denoted by Roman numerals. The short line indicated the inferred Watson-Crick bonds, and the dark dots indicated GU bonds.

The mt rRNA genes have frequently not been annotated via the use of functional features, so it is hard to annotate them from their DNA sequences alone [56, 60–61]. Beckenbach has proposed that the start of *srRNA* is AARGUUUU based on an alignment across dipteran and mecopteran sequences [6]. Hence, we annotated the *lrRNA* gene as in other dipteran species, where it is between *tRNA*^*Leu (CUN)*^ and *tRNA*^*Val*^, while the *srRNA* gene is flanked at the 3’ end by *tRNA*^*Val*^ and the motif AARGUUUU. Furthermore, we inferred the secondary structures for *lrRNA* and *srRNA* in the Tipuloidea using the sequences of *S*. *hybrida* based on the published *lrRNA* and *srRNA* secondary structures, the sepsid fly *Nemopoda mamaevi* Ozerov, 1997 [56]. The secondary structures of *lrRNA* and *srRNA* are similar to those in *N*. *mamaevi* and other Dipteran species [56, 62]. The *lrRNA* has five structural domains (domain III absent as in other insects) and 42 helices while the *srRNA* includes 3 domains and 25 helices (Figs 4 and 5).

### Phylogeny of Tipulomorpha

**Fig 6:**
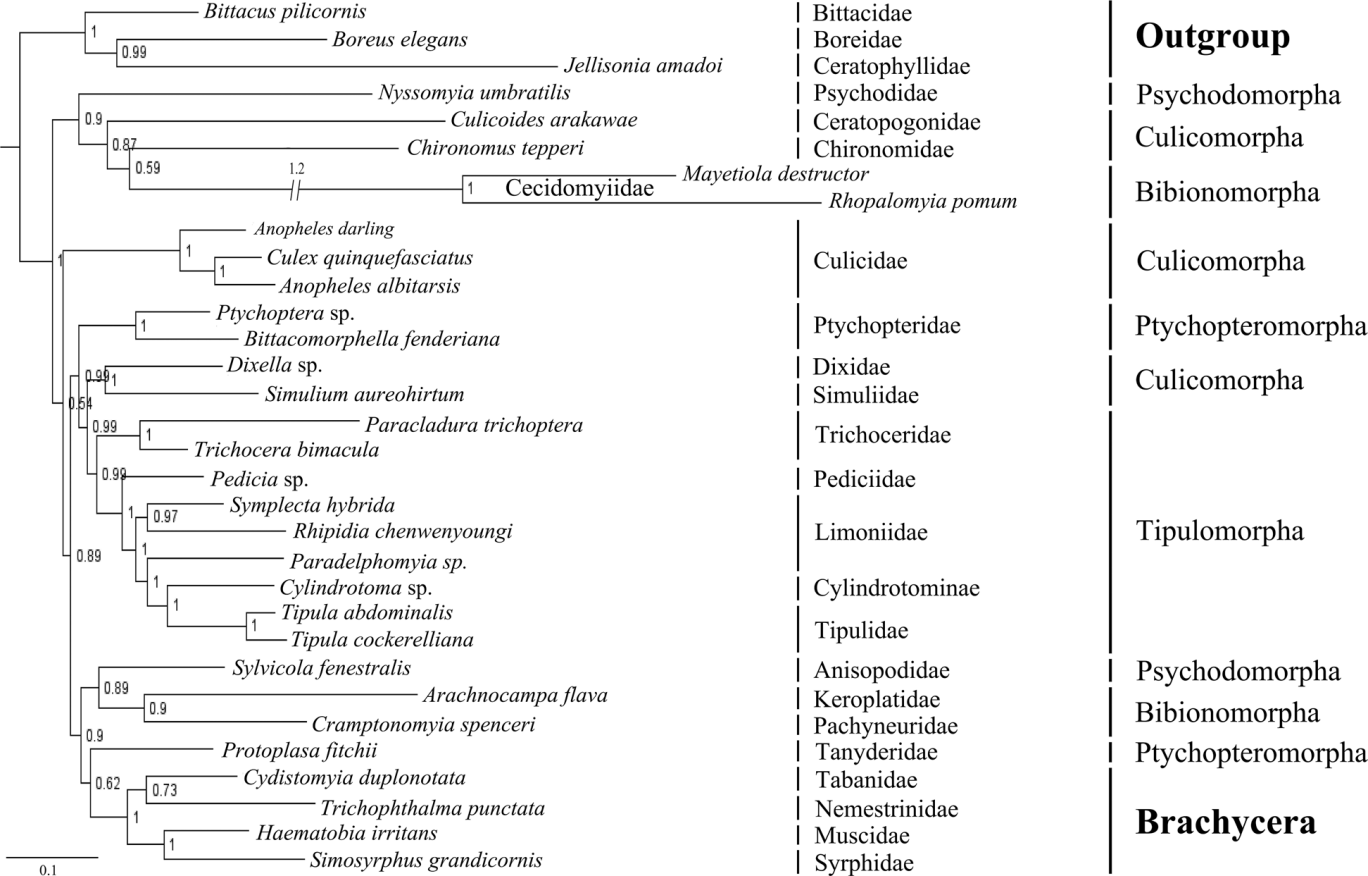
Phylogenetic tree of Nematocera based on mt genome data PCG12RNA. Cladogram of relationships resulting from BI with *Bittacus pilicornis*, *Boreus elegans* and *Jellisonia amadoi* as outgroups. Numbers above the branches are posterior probabilities.

**Fig 7:**
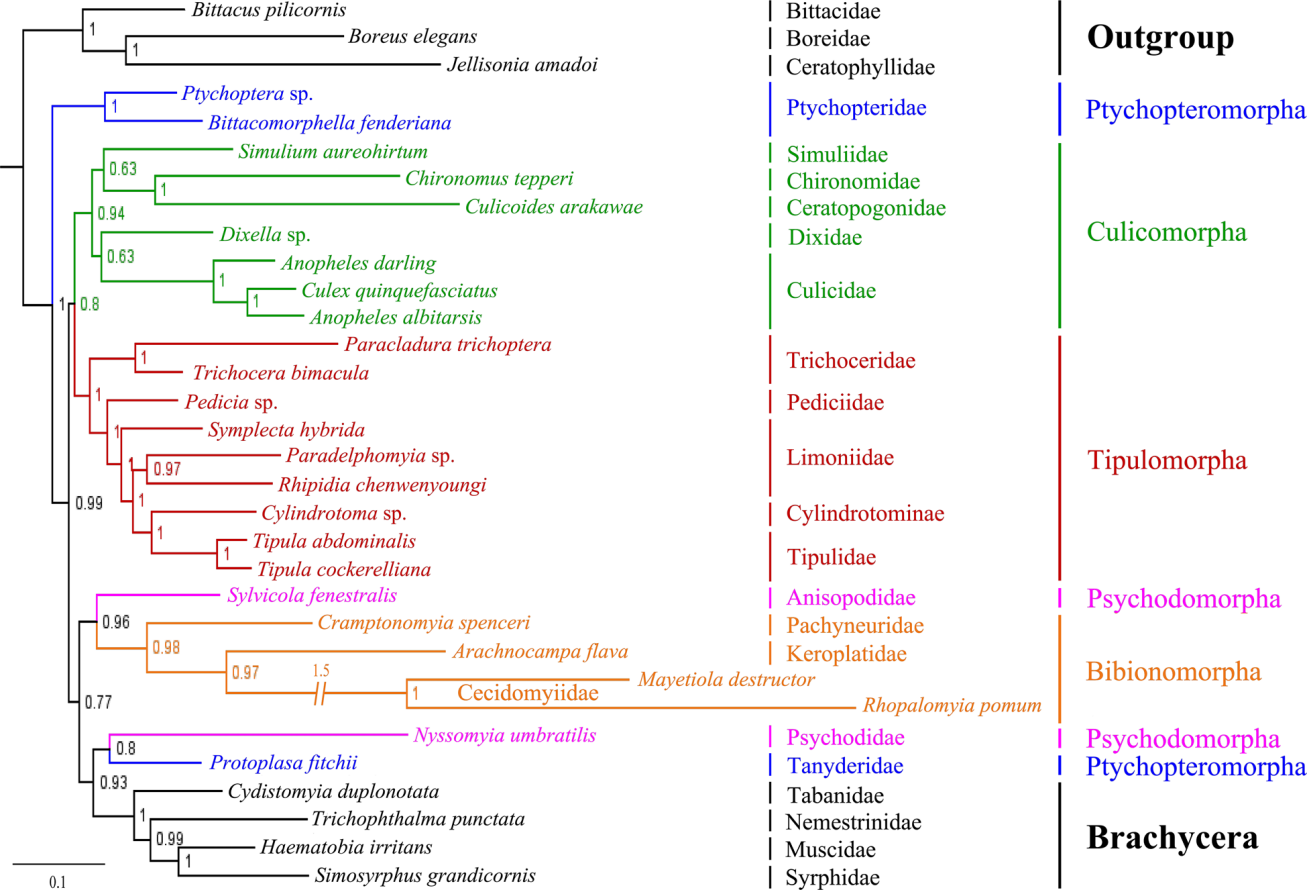
Phylogenetic tree of Nematocera based on mt genome data 5PCG12RNA. Cladogram of relationships resulting from BI with *Bittacus pilicornis*, *Boreus elegans* and *Jellisonia amadoi* as outgroups. Numbers above the branches are posterior probabilities.

The phylogenetic trees based on BI analyses of two datasets are given in Figs 6 and 7. The tree based on dataset PCG12RNA is disordered in four main branches (Fig 6), especially in the infraorder Culicomorpha, which was a well-supported monophyletic clade in previous studies [26, 29, 30, 41, 63, 64]. Then, we construct another tree based on dataset 5PCG12RNA, which are excluded eight difficult aligned genes (Fig 7). The monophyly of infraorder Culicomorpha is well supported, as well as the Tipulomorpha and the Bibionomorpha. The BI tree of 5PCG12RNA supports the Ptychopteromorpha is the earliest branch within the Diptera, and Psychodidae+Tanyderidae is the sister group to the Brachycera. However, two phylogenetic trees have very similar topologies for the branch Tipulomorpha. The monophyly of Tipulomorpha (Trichoceridae + Tipuloidea) is consistently supported, as is the monophyly of Tipuloidea (Cylindrotomidae, Limoniidae, Pediciidae, and Tipulidae *sensu stricto*). The monophyly of the family Limoniidae is not supported, with one or more of the three limoniid species grouping sister to the clade Cylindrotomidae + Tipulidae in each analyses. All analyses support the Tipulomorpha as having an intermediate phylogenetic position within the lower Diptera, never sister to the remaining flies or to the derived Brachycera.

The earliest linkage of the Nematocera and the phylogenetic position of the Tipulomorpha within the lower Diptera were controversial. Tipulomorpha has been inferred to be the earliest lineage of the Nematocera [26–27, 30] or as the most derived branch in the Nematocera [24]. Transcriptome-based phylogenetic studies, however, thought that the Culicomorpha as the earliest branch of the Diptera with the Tipulomorpha in intermediate branch [42–43]. Our BI tree of 5PCG12RNA supports the Ptychopteromorpha as the earliest branch of the Diptera. This result is consistent with Oosterbroek and Courtney’s morphological findings [31]. Bertone’s phylogenetic tree based on multiple nuclear genes also supports the Ptychopteromorpha topologically as one of the earliest branch of the order [38]. For the position of the Tipulomorpha, all analyses in our study support it as having an intermediate phylogenetic position within the Nematocera, never sister to the remaining flies or to the derived Brachycera.

The composition of the infraorder Tipulomorpha has long been contentious. It has been variously defined to include both Tipuloidea and Trichoceridae or just Tipuloidea [25–30]. Our molecular data supports a more traditional conception of Tipulomorpha as containing both Tipuloidea and Trichoceridae, consistent with Hennig’s hypothesis [26]. This relationship has also been accepted by some other researchers [35–39]. Beckenbach’s mt genome phylogeny, however, failed to give a clear resolution of this question [6]. In Beckenbach’s study, one analysis using only a set of less variable major genes (*CO1*, *CO2*, *CO3*, *CytB*, *ATP6* and rRNAs) supported the pairing of these two families, whereas inclusion of all major genes inferred a topology that would define Tipulomorpha as only consisting of Tipuloidea.

Within the Tipuloidea, Starý (1992) considered that the Limoniidae was the sister-group to a clade Pediciidae + (Tipulidae + Cylindrotomidae) according to 11 adult morphological characters [65]. The arrangement of Pediciidae being the sister-group to the remaining Tipuloidea, was accepted by Ribeiro (2008) on the basis of 88 morphological characters and by Petersen *et al*. (2010) based on combined morphological characters and two nuclear genes. Petersen *et al*. (2010) showed that Cylindrotomidae and Tipulidae were sister-group. However, their placement within Tipuloidea was less certain [66–67]. In our study, both BI analyses support the Pediciidae as the sister-group of the remaining Tipuloidea. The sister relationship between Tipulidae and Cylindrotomidae is also strongly supported in both analyses. These results are concordant with Petersen *et al*.’s research, which presented a new classification system recognizing a two-family Tipuloidea (Tipulidae and Pediciidae) [67]. Two trees had different topologies across Limoniidae, with the Limnophilinae sister to Chioneinae + Limoniinae and the Chioneinae sister to Limnophilinae + Limoniinae. Anyway, family Limoniidae is not supported as a monophyletic clade and subfamily Limoniinae seems to have a closer relationship with Cylindrotomidae + Tipulidae.

Compared with Beckenbach’s study, we come to a steady conclusion on the composition of the infraorder Tipulomorpha. The variation of the composition the Tipulomorpha in Beckenbach’s study might be caused by lack of data from other tipuloids, especially the family Pediciidae, which was the sister of the remaining Tipuloidea, in our BI analyses, or in a clade (along with members of the Limoniidae) that is sister to the remaining Tipuloidea. Therefore, we consider that increasing the sampling comprehensiveness, especially the relatively primitive group, can help to give us a more reasonable phylogenetic tree.

In our study, Limoniidae is not a monophyletic clade. The family Limoniidae consists of four subfamilies proposed by Starý (1992), of which the subfamily Dactylolabinae contains only one genus. Although we selected representatives for the remaining three subfamilies, it seems that our current taxon sampling is not extensive enough to build an initial framework of these clades. Therefore, further detailed studies with more taxa are needed before natural families can be confidently defined within the Tipuloidea.

## Supporting Information

S1 Table Collection information of specimens. (DOCX)

S2 Table Primers used in this study. (DOCX)

S3 Table The best partitioning scheme selected by PartitionFinder for different dataset. (DOCX)

S4 Table Intergenic sequence in all sequenced flies of Tipuloidea. (DOCX)

S5 Table Codon usage of Tipuloidea mt genomes. (DOCX)

S6 Table Usage of start and stop codons in Nematocera mt genomes. (XLSX)
