# Supplementary material for: Comparative Mt Genomics of the Tipuloidea (Diptera: Nematocera: Tipulomorpha) and Its Implications for the Phylogeny of the Tipulomorpha
Source: PLoS One. 2016 Jun 24;11(6):e0158167. doi: 10.1371/journal.pone.0158167 (PMC4920351; doi:10.1371/journal.pone.0158167)
Supplement: S1 Table — (DOCX) [file pone.0158167.s001.docx]

**S1 Table** Collection information of speciman

| **Species** | **Collection Location** | **Collection Data** | **Collectors** |
| --- | --- | --- | --- |
| *Tipula cockerelliana* | China, Shannxi, Meixian, Mt. Taibai (1177m) | 2013.VIII.24 | Yuqiang Xi |
| *Symplecta hybrida* | China, Neimenggu, Tongliao, Daqinggou | 2014.VIII.20 | Yuqiang Xi |
| *Rhipidia chenwenyoungi* | China, Taiwan, Nantou, Lianhuachi | 2013.VI.5 | Wenliang Li |
| *Paradelphomyia* sp. | China, Guangxi, Nanning, Mt. Daming (1230m) | 2013.V.21 | Yuqiang Xi |
| *Cylindrotoma* sp. | China, Yunnan, Baoshan | 2012.V.10 | Wenliang Li |
| *Pedicia* sp. | China, Neimenggu, Tongliao, Daqinggou | 2014.VIII.20 | Yan Li |
