# Supplementary material for: Comparative Mt Genomics of the Tipuloidea (Diptera: Nematocera: Tipulomorpha) and Its Implications for the Phylogeny of the Tipulomorpha
Source: PLoS One. 2016 Jun 24;11(6):e0158167. doi: 10.1371/journal.pone.0158167 (PMC4920351; doi:10.1371/journal.pone.0158167)
Supplement: S2 Table — (DOCX) [file pone.0158167.s002.docx]

**S2 Table.** Primers used in this study.

| Number | Primer pairs (F/R) | Sequence (forward and reverse) 5’-3’ | Size (bp) |
| --- | --- | --- | --- |
| 1 | FL34/TR1284 | GCCTGAAAAAGGGTTACTTTGATA/ACARCTTTGAAGGYTAWTAGTTT | 1200 |
| 2 | F20/R20 | CCATTCCATTTYTGATTTCC/ TTTATTCGTGGAAATGCTATGTC | 1100 |
| 3 | F21/R21 | AATTGGTGGTTTTGGAAATTG/GGTAATCAGAGTATCGACG | 1000 |
| 4 | F22/R22 | ACATTTTTTCCTCAACATTT/ TATTCATATCTTCAATATCATTGATG | 600 |
| 5 | F01/R01 | ACATTTTTTCCTCAACATTT/ CCACAAATTTCTGAACACTG | 900 |
| 6 | F02/R02 | TCTATTGGTCATCAATGGTACTG/ GAAAATAAATTTGTTATCATTTTCA | 600 |
| 7 | F03/R03 | CATTAAGTGACTGAAAGCAAGTA/ ATGACCTGCAATTATATTAGC | 700 |
| 8 | TF4463/ TR4908 | TTTGCCCATCTWGTWCCNCAAGG/ CGAGTTAYATCTCGTCATCATTG | 500 |
| 9 | F05/R05 | GTAGATGCAAGCCCTTGACC/ ATTGGATCAAATCCACATTC | 900 |
| 10 | TF5747/TR6384 | CCATTTGAATGTGGRTTTGAYCC/ TATATTTAGAGYATRAYAYTGAAG | 600 |
| 11 | TF6400/TR7211 | TAACATCTTCAATRTYATRCTCT/ TTAAGGCTTTAYTATTTATRTGYGC | 800 |
| 12 | F08/R08 | TTAAATCCTTTGAGTAAAATCC/TTAGGTTGAGATGGTTTAGG | 700 |
| 13 | TF7806/TR8727 | GAMACAARACCTAACCCATCYCA/AAATCTTTRATTGCTTATTCWTC | 900 |
| 14 | F09/R09 | AAACGGAAACTGAGCTCTCTTAGT/ AAATCTTTAATTGCCTATTCTTC | 800 |
| 15 | TF8641/TR9153 | CCAGAAGAACATAANCCRTG/ TGAGGTTATCAACCNGARCG | 500 |
| 16 | TF8941/TR9629 | GAAACAGGAGCCTCAACATGWGC/ GTTTGTGAGGGWGYTTTRGG | 700 |
| 17 | TF9172/TR10608 | CGCTCAGGYTGRTACCCYCA/ CCAAGTARTGAWCCAAARTTTCA | 1400 |
| 18 | TF9648/TR11010 | ACCTAAAGCTCCCTCACAWAC/TATCTACAGCRAATCCYCCYCA | 1300 |
| 19 | F23/R23 | CTCATACTGATGAAATTTTGGTTC/TTCTACTGGTCGTGCTCCAATTCA | 900 |
| 20 | F12/R12 | CATATTCAACCAGAATGATA/ AATCGTTCTCCATTTGATTTTGC | 700 |
| 21 | F13/R13 | CGAGGTAAAGTACCACGTACTCA/ GTTGGATTTCTAACTTTATTRGARCG | 700 |
| 22 | F15/R15 | CCGGTCTGAACTCAGATCATGTA/ ATTTATTGTACCTTTTGTATCAG | 1000 |
| 23 | F16/R16 | CCTTTGCACAGTCAAAATACTGC/ TTATGCACACATCGCCCGTC | 900 |
| 24 | F17/R17 | GTAAAYCTACTTTGTTACGACTT/ GTGCCAGCAAYCGCGGTTATAC | 600 |
