# Supplementary material for: Comparative Mt Genomics of the Tipuloidea (Diptera: Nematocera: Tipulomorpha) and Its Implications for the Phylogeny of the Tipulomorpha
Source: PLoS One. 2016 Jun 24;11(6):e0158167. doi: 10.1371/journal.pone.0158167 (PMC4920351; doi:10.1371/journal.pone.0158167)
Supplement: S3 Table — (DOCX) [file pone.0158167.s003.docx]

**S3 Table.** The best partitioning scheme selected by PartitionFinder for different dataset.

| **Dataset** | **Subset Partitions** | **Best Model** |
| --- | --- | --- |
| PCG12RNA:  5 partitions (BI) | P1: (ATP6_pos1, CO1_pos1, CO2_pos1, CO3_pos1, cytB_pos1, tRNA_NT) | GTR+I+G |
|  | P2: (ATP6_pos2, CO1_pos2, CO2_pos2, CO3_pos2, ND3_pos2, cytB_pos2) | GTR+I+G |
|  | P3: (ATP8_pos1, ATP8_pos2, ND2_pos1, ND2_pos2, ND3_pos1, ND6_pos1, ND6_pos2) | GTR+I+G |
|  | P4: (ND1_pos1, ND4L_pos1, ND4L_pos2, ND4_pos1, ND5_pos1) | GTR+I+G |
|  | P5: (ND1_pos2, ND4_pos2, ND5_pos2, 12S_NT, 16S_NT) | GTR+I+G |
| 5PCG12RNA:  3 partitions (BI) | P1: (ATP6_pos1, CO1_pos1, CO2_pos1, CO3_pos1, cytB_pos1, tRNA_NT) | GTR+I+G |
|  | P2: (ATP6_pos2, CO1_pos2, CO2_pos2, CO3_pos2, ND3_pos2, cytB_pos2) | GTR+I+G |
|  | P3: (12S_NT, 16S_NT) | GTR+I+G |
