# Supplementary material for: Comparative Mt Genomics of the Tipuloidea (Diptera: Nematocera: Tipulomorpha) and Its Implications for the Phylogeny of the Tipulomorpha
Source: PLoS One. 2016 Jun 24;11(6):e0158167. doi: 10.1371/journal.pone.0158167 (PMC4920351; doi:10.1371/journal.pone.0158167)
Supplement: S4 Table — (DOCX) [file pone.0158167.s004.docx]

| **Species** | **Intergenic Sequence between *tRNA^Glu^* and *tRNA^Phe^*** | **No.** | **Intergenic Sequence between *tRNA^Ser(UCN)^* and *ND1*** | **No.** |
| --- | --- | --- | --- | --- |
| *Symplecta hybrida* | ATTGCTATAATTAGTTATTATAAATAAATA | 30 | TACTAATAATAATTAACTA | 19 |
| *Rhipidia chenwenyoungi* | TACAATAATTAATTATTTAATAATTATACTAT | 32 | TACTATTTTATATTCA | 16 |
| *Paradelphomyia* sp. | ACTAAATTATATTATTTAAT | 20 | ACTTACTTAAACTTTTTAAATTTACTAAAAATTATTAT | 38 |
| *Cylindrotoma* sp. | ACTAAAATTAGTTATTTAA | 19 | TACTAATTTATATTAA | 16 |
| *Pedicia* sp. | ACTAAAACATGTTCATCAA | 19 | TACTAAATTATATTAA | 16 |
| *Tipula cockerelliana* | ACTACTTTTTATTATACAAATTAATTA | 27 | TACTAATATTAATGAT | 16 |
