# Supplementary material for: Comparative Mt Genomics of the Tipuloidea (Diptera: Nematocera: Tipulomorpha) and Its Implications for the Phylogeny of the Tipulomorpha
Source: PLoS One. 2016 Jun 24;11(6):e0158167. doi: 10.1371/journal.pone.0158167 (PMC4920351; doi:10.1371/journal.pone.0158167)
Supplement: S5 Table — (DOCX) [file pone.0158167.s005.docx]

S5 Table. Codon usage of the *Symplecta hybrida* mt genome.

| Amino acid | Codon | N | RSCU | N+ | RSCU | N- | RSCU |
| --- | --- | --- | --- | --- | --- | --- | --- |
| Phe(F) | UUU(F) | 307 | 1.8 | 175 | 1.71 | 132 | 1.94 |
|  | UUC(F) | 34 | 0.2 | 30 | 0.29 | 4 | 0.06 |
| Leu^UUR^ (L) | UUA(L) | 499 | 4.84 | 257 | 4.41 | 242 | 5.42 |
|  | UUG(L) | 29 | 0.28 | 15 | 0.26 | 14 | 0.31 |
| Leu^CUN^ (L) | CUU(L) | 56 | 0.54 | 50 | 0.86 | 6 | 0.13 |
|  | CUC(L) | 5 | 0.05 | 5 | 0.09 | 0 | 0 |
|  | CUA(L) | 27 | 0.26 | 21 | 0.36 | 6 | 0.13 |
|  | CUG(L) | 2 | 0.02 | 2 | 0.03 | 0 | 0 |
| Ile (I) | AUU(I) | 309 | 1.91 | 193 | 1.88 | 116 | 1.95 |
|  | AUC(I) | 15 | 0.09 | 12 | 0.12 | 3 | 0.05 |
| Met (M) | AUA(M) | 198 | 1.77 | 104 | 1.72 | 94 | 1.83 |
|  | AUG(M) | 26 | 0.23 | 17 | 0.28 | 9 | 0.17 |
| Val (V) | GUU(V) | 101 | 2.06 | 50 | 1.69 | 51 | 2.62 |
|  | GUC(V) | 7 | 0.14 | 6 | 0.2 | 1 | 0.05 |
|  | GUA(V) | 80 | 1.63 | 57 | 1.93 | 23 | 1.18 |
|  | GUG(V) | 8 | 0.16 | 5 | 0.17 | 3 | 0.15 |
| Ser^UCN^ (S) | UCU(S) | 101 | 2.67 | 51 | 2.5 | 50 | 2.86 |
|  | UCC(S) | 8 | 0.21 | 6 | 0.29 | 2 | 0.11 |
|  | UCA(S) | 87 | 2.3 | 61 | 2.99 | 26 | 1.49 |
|  | UCG(S) | 7 | 0.18 | 7 | 0.34 | 0 | 0 |
| Pro (P) | CCU(P) | 85 | 2.54 | 61 | 2.52 | 24 | 2.59 |
|  | CCC(P) | 4 | 0.12 | 4 | 0.16 | 0 | 0 |
|  | CCA(P) | 43 | 1.28 | 31 | 1.28 | 12 | 1.3 |
|  | CCG(P) | 2 | 0.06 | 1 | 0.04 | 1 | 0.11 |
| Thr (T) | ACU(T) | 80 | 1.85 | 56 | 1.76 | 24 | 2.09 |
|  | ACC(T) | 7 | 0.16 | 6 | 0.19 | 1 | 0.09 |
|  | ACA(T) | 82 | 1.9 | 61 | 1.92 | 21 | 1.83 |
|  | ACG(T) | 4 | 0.09 | 4 | 0.13 | 0 | 0 |
| Ala (A) | GCU(A) | 126 | 2.72 | 73 | 2.5 | 53 | 3.12 |
|  | GCC(A) | 17 | 0.37 | 15 | 0.51 | 2 | 0.12 |
|  | GCA(A) | 41 | 0.89 | 29 | 0.99 | 12 | 0.71 |
|  | GCG(A) | 1 | 0.02 | 0 | 0 | 1 | 0.06 |
| Tyr (Y) | UAU(Y) | 156 | 1.77 | 82 | 1.64 | 74 | 1.95 |
|  | UAC(Y) | 20 | 0.23 | 18 | 0.36 | 2 | 0.05 |
| Stop (*) | UAA(*) | 38 | 1.52 | 38 | 1.52 | 0 | 0 |
|  | UAG(*) | 12 | 0.48 | 12 | 0.48 | 0 | 0 |
| His (H) | CAU(H) | 73 | 1.7 | 62 | 1.68 | 11 | 1.83 |
|  | CAC(H) | 13 | 0.3 | 12 | 0.32 | 1 | 0.17 |
| Gln (Q) | CAA(Q) | 72 | 1.89 | 51 | 1.89 | 21 | 1.91 |
|  | CAG(Q) | 4 | 0.11 | 3 | 0.11 | 1 | 0.09 |
| Asn (N) | AAU(N) | 175 | 1.89 | 105 | 1.83 | 70 | 2 |
|  | AAC(N) | 10 | 0.11 | 10 | 0.17 | 0 | 0 |
| Lys (K) | AAA(K) | 71 | 1.48 | 42 | 1.56 | 29 | 1.38 |
|  | AAG(K) | 25 | 0.52 | 12 | 0.44 | 13 | 0.62 |
| Asp (D) | GAU(D) | 59 | 1.62 | 38 | 1.49 | 21 | 1.91 |
|  | GAC(D) | 14 | 0.38 | 13 | 0.51 | 1 | 0.09 |
| Glu (E) | GAA(E) | 78 | 1.9 | 45 | 1.88 | 33 | 1.94 |
|  | GAG(E) | 4 | 0.1 | 3 | 0.13 | 1 | 0.06 |
| Cys (C) | UGU(C) | 38 | 1.9 | 12 | 1.85 | 26 | 1.93 |
|  | UGC(C) | 2 | 0.1 | 1 | 0.15 | 1 | 0.07 |
| Trp (W) | UGA(W) | 93 | 1.82 | 68 | 1.92 | 25 | 1.61 |
|  | UGG(W) | 9 | 0.18 | 3 | 0.08 | 6 | 0.39 |
| Arg (R) | CGU(R) | 9 | 0.67 | 2 | 0.24 | 7 | 1.4 |
|  | CGC(R) | 0 | 0 | 0 | 0 | 0 | 0 |
|  | CGA(R) | 42 | 3.11 | 32 | 3.76 | 10 | 2 |
|  | CGG(R) | 3 | 0.22 | 0 | 0 | 3 | 0.6 |
| Ser^AGN^ (S) | AGU(S) | 52 | 1.37 | 22 | 1.08 | 30 | 1.71 |
|  | AGC(S) | 4 | 0.11 | 2 | 0.1 | 2 | 0.11 |
|  | AGA(S) | 44 | 1.16 | 14 | 0.69 | 30 | 1.71 |
|  | AGG(S) | 0 | 0 | 0 | 0 | 0 | 0 |
| Gly (G) | GGU(G) | 38 | 0.71 | 19 | 0.6 | 19 | 0.88 |
|  | GGC(G) | 4 | 0.08 | 4 | 0.13 | 0 | 0 |
|  | GGA(G) | 132 | 2.48 | 92 | 2.9 | 40 | 1.86 |
|  | GGG(G) | 39 | 0.73 | 12 | 0.38 | 27 | 1.26 |

Codon usage of the *Pedicia* sp mt genome.

| Amino acid | Codon | N | RSCU | N+ | RSCU | N- | RSCU |
| --- | --- | --- | --- | --- | --- | --- | --- |
| Phe(F) | UUU(F) | 304 | 1.77 | 155 | 1.62 | 149 | 1.96 |
|  | UUC(F) | 39 | 0.23 | 36 | 0.38 | 3 | 0.04 |
| Leu^UUR^ (L) | UUA(L) | 332 | 3.51 | 173 | 3.22 | 159 | 3.88 |
|  | UUG(L) | 76 | 0.8 | 13 | 0.24 | 63 | 1.54 |
| Leu^CUN^ (L) | CUU(L) | 58 | 0.61 | 38 | 0.71 | 20 | 0.49 |
|  | CUC(L) | 6 | 0.06 | 6 | 0.11 | 0 | 0 |
|  | CUA(L) | 86 | 0.91 | 82 | 1.53 | 4 | 0.1 |
|  | CUG(L) | 10 | 0.11 | 10 | 0.19 | 0 | 0 |
| Ile (I) | AUU(I) | 298 | 1.72 | 197 | 1.64 | 101 | 1.88 |
|  | AUC(I) | 27 | 0.16 | 24 | 0.2 | 3 | 0.06 |
| Met (M) | AUA(M) | 196 | 1.13 | 139 | 1.16 | 57 | 1.06 |
|  | AUG(M) | 54 | 1 | 12 | 1 | 42 | 1 |
| Val (V) | GUU(V) | 116 | 2.04 | 40 | 1.23 | 76 | 3.13 |
|  | GUC(V) | 10 | 0.18 | 7 | 0.22 | 3 | 0.12 |
|  | GUA(V) | 92 | 1.62 | 80 | 2.46 | 12 | 0.49 |
|  | GUG(V) | 9 | 0.16 | 3 | 0.09 | 6 | 0.25 |
| Ser^UCN^ (S) | UCU(S) | 121 | 2.65 | 52 | 1.99 | 69 | 3.54 |
|  | UCC(S) | 21 | 0.46 | 18 | 0.69 | 3 | 0.15 |
|  | UCA(S) | 75 | 1.64 | 66 | 2.52 | 9 | 0.46 |
|  | UCG(S) | 8 | 0.18 | 5 | 0.19 | 3 | 0.15 |
| Pro (P) | CCU(P) | 55 | 1.62 | 27 | 1.08 | 28 | 3.11 |
|  | CCC(P) | 12 | 0.35 | 10 | 0.4 | 2 | 0.22 |
|  | CCA(P) | 60 | 1.76 | 58 | 2.32 | 2 | 0.22 |
|  | CCG(P) | 9 | 0.26 | 5 | 0.2 | 4 | 0.44 |
| Thr (T) | ACU(T) | 96 | 2.11 | 59 | 1.7 | 37 | 3.44 |
|  | ACC(T) | 19 | 0.42 | 19 | 0.55 | 0 | 0 |
|  | ACA(T) | 65 | 1.43 | 59 | 1.7 | 6 | 0.56 |
|  | ACG(T) | 2 | 0.04 | 2 | 0.06 | 0 | 0 |
| Ala (A) | GCU(A) | 96 | 2.01 | 49 | 1.58 | 47 | 2.81 |
|  | GCC(A) | 32 | 0.67 | 30 | 0.97 | 2 | 0.12 |
|  | GCA(A) | 56 | 1.17 | 42 | 1.35 | 14 | 0.84 |
|  | GCG(A) | 7 | 0.15 | 3 | 0.1 | 4 | 0.24 |
| Tyr (Y) | UAU(Y) | 132 | 1.59 | 56 | 1.3 | 76 | 1.9 |
|  | UAC(Y) | 34 | 0.41 | 30 | 0.7 | 4 | 0.1 |
| Stop (*) | UAA(*) | 0 | 0 | 0 | 0 | 0 | 0 |
|  | UAG(*) | 0 | 0 | 0 | 0 | 0 | 0 |
| His (H) | CAU(H) | 49 | 1.21 | 35 | 1.04 | 14 | 2 |
|  | CAC(H) | 32 | 0.79 | 32 | 0.96 | 0 | 0 |
| Gln (Q) | CAA(Q) | 64 | 1.78 | 48 | 1.96 | 16 | 1.39 |
|  | CAG(Q) | 8 | 0.22 | 1 | 0.04 | 7 | 0.61 |
| Asn (N) | AAU(N) | 145 | 1.55 | 81 | 1.32 | 64 | 2 |
|  | AAC(N) | 42 | 0.45 | 42 | 0.68 | 0 | 0 |
| Lys (K) | AAA(K) | 63 | 1.5 | 43 | 1.79 | 20 | 1.11 |
|  | AAG(K) | 21 | 0.5 | 5 | 0.21 | 16 | 0.89 |
| Asp (D) | GAU(D) | 49 | 1.44 | 28 | 1.22 | 21 | 1.91 |
|  | GAC(D) | 19 | 0.56 | 18 | 0.78 | 1 | 0.09 |
| Glu (E) | GAA(E) | 66 | 1.65 | 44 | 1.91 | 22 | 1.29 |
|  | GAG(E) | 14 | 0.35 | 2 | 0.09 | 12 | 0.71 |
| Cys (C) | UGU(C) | 37 | 1.68 | 8 | 1.07 | 29 | 2 |
|  | UGC(C) | 7 | 0.32 | 7 | 0.93 | 0 | 0 |
| Trp (W) | UGA(W) | 93 | 3 | 69 | 3 | 24 | 3 |
|  | UGG(W) | 10 | 1 | 2 | 1 | 8 | 1 |
| Arg (R) | CGU(R) | 14 | 0.67 | 4 | 0.33 | 10 | 1.13 |
|  | CGC(R) | 1 | 0.05 | 0 | 0 | 1 | 0.11 |
|  | CGA(R) | 42 | 2.02 | 33 | 2.75 | 9 | 1.02 |
|  | CGG(R) | 1 | 0.05 | 0 | 0 | 1 | 0.11 |
| Ser^AGN^ (S) | AGU(S) | 44 | 0.96 | 13 | 0.5 | 31 | 1.59 |
|  | AGC(S) | 5 | 0.11 | 3 | 0.11 | 2 | 0.1 |
|  | AGA(S) | 66 | 3.17 | 35 | 2.92 | 31 | 3.51 |
|  | AGG(S) | 1 | 0.05 | 0 | 0 | 1 | 0.11 |
| Gly (G) | GGU(G) | 52 | 0.93 | 9 | 0.27 | 43 | 1.93 |
|  | GGC(G) | 7 | 0.13 | 5 | 0.15 | 2 | 0.09 |
|  | GGA(G) | 121 | 2.16 | 102 | 3.02 | 19 | 0.85 |
|  | GGG(G) | 44 | 0.79 | 19 | 0.56 | 25 | 1.12 |

Codon usage of the *Tipula cockerelliana* mt genome.

| Amino acid | Codon | N | RSCU | N+ | RSCU | N- | RSCU |
| --- | --- | --- | --- | --- | --- | --- | --- |
| Phe(F) | UUU(F) | 325 | 1.74 | 172 | 1.75 | 153 | 1.74 |
|  | UUC(F) | 48 | 0.26 | 25 | 0.25 | 23 | 0.26 |
| Leu^UUR^ (L) | UUA(L) | 349 | 3.85 | 213 | 3.64 | 136 | 4.23 |
|  | UUG(L) | 52 | 0.57 | 12 | 0.21 | 40 | 1.24 |
| Leu^CUN^ (L) | CUU(L) | 72 | 0.79 | 65 | 1.11 | 7 | 0.22 |
|  | CUC(L) | 12 | 0.13 | 11 | 0.19 | 1 | 0.03 |
|  | CUA(L) | 58 | 0.64 | 49 | 0.84 | 9 | 0.28 |
|  | CUG(L) | 1 | 0.01 | 1 | 0.02 | 0 | 0 |
| Ile (I) | AUU(I) | 324 | 1.86 | 207 | 1.76 | 117 | 2.08 |
|  | AUC(I) | 44 | 0.25 | 35 | 0.3 | 9 | 0.16 |
| Met (M) | AUA(M) | 154 | 0.89 | 111 | 0.94 | 43 | 0.76 |
|  | AUG(M) | 35 | 1 | 9 | 1 | 26 | 1 |
| Val (V) | GUU(V) | 65 | 1.41 | 30 | 1.1 | 35 | 1.84 |
|  | GUC(V) | 19 | 0.41 | 14 | 0.51 | 5 | 0.26 |
|  | GUA(V) | 87 | 1.88 | 61 | 2.24 | 26 | 1.37 |
|  | GUG(V) | 14 | 0.3 | 4 | 0.15 | 10 | 0.53 |
| Ser^UCN^ (S) | UCU(S) | 89 | 1.94 | 58 | 2.11 | 31 | 1.69 |
|  | UCC(S) | 27 | 0.59 | 18 | 0.65 | 9 | 0.49 |
|  | UCA(S) | 75 | 1.64 | 57 | 2.07 | 18 | 0.98 |
|  | UCG(S) | 14 | 0.31 | 3 | 0.11 | 11 | 0.6 |
| Pro (P) | CCU(P) | 45 | 1.4 | 29 | 1.13 | 16 | 2.46 |
|  | CCC(P) | 38 | 1.18 | 35 | 1.36 | 3 | 0.46 |
|  | CCA(P) | 44 | 1.36 | 37 | 1.44 | 7 | 1.08 |
|  | CCG(P) | 2 | 0.06 | 2 | 0.08 | 0 | 0 |
| Thr (T) | ACU(T) | 72 | 1.65 | 57 | 1.57 | 15 | 2 |
|  | ACC(T) | 29 | 0.66 | 26 | 0.72 | 3 | 0.4 |
|  | ACA(T) | 73 | 1.67 | 62 | 1.71 | 11 | 1.47 |
|  | ACG(T) | 1 | 0.02 | 0 | 0 | 1 | 0.13 |
| Ala (A) | GCU(A) | 75 | 1.84 | 47 | 1.54 | 28 | 2.73 |
|  | GCC(A) | 33 | 0.81 | 28 | 0.92 | 5 | 0.49 |
|  | GCA(A) | 47 | 1.15 | 42 | 1.38 | 5 | 0.49 |
|  | GCG(A) | 8 | 0.2 | 5 | 0.16 | 3 | 0.29 |
| Tyr (Y) | UAU(Y) | 160 | 1.7 | 65 | 1.48 | 95 | 1.9 |
|  | UAC(Y) | 28 | 0.3 | 23 | 0.52 | 5 | 0.1 |
| Stop (*) | UAA(*) | 25 | 0.61 | 0 | 0 | 25 | 1.29 |
|  | UAG(*) | 10 | 0.24 | 0 | 0 | 10 | 0.52 |
| His (H) | CAU(H) | 46 | 1.26 | 39 | 1.18 | 7 | 2 |
|  | CAC(H) | 27 | 0.74 | 27 | 0.82 | 0 | 0 |
| Gln (Q) | CAA(Q) | 60 | 1.82 | 48 | 1.92 | 12 | 1.5 |
|  | CAG(Q) | 6 | 0.18 | 2 | 0.08 | 4 | 0.5 |
| Asn (N) | AAU(N) | 183 | 1.65 | 103 | 1.6 | 80 | 1.72 |
|  | AAC(N) | 39 | 0.35 | 26 | 0.4 | 13 | 0.28 |
| Lys (K) | AAA(K) | 81 | 1.6 | 42 | 1.87 | 39 | 1.39 |
|  | AAG(K) | 20 | 0.4 | 3 | 0.13 | 17 | 0.61 |
| Asp (D) | GAU(D) | 41 | 1.41 | 30 | 1.33 | 11 | 1.69 |
|  | GAC(D) | 17 | 0.59 | 15 | 0.67 | 2 | 0.31 |
| Glu (E) | GAA(E) | 63 | 1.68 | 46 | 1.92 | 17 | 1.26 |
|  | GAG(E) | 12 | 0.32 | 2 | 0.08 | 10 | 0.74 |
| Cys (C) | UGU(C) | 43 | 1.51 | 10 | 1.54 | 33 | 1.5 |
|  | UGC(C) | 14 | 0.49 | 3 | 0.46 | 11 | 0.5 |
| Trp (W) | UGA(W) | 88 | 2.15 | 65 | 3 | 23 | 1.19 |
|  | UGG(W) | 24 | 1 | 5 | 1 | 19 | 1 |
| Arg (R) | CGU(R) | 9 | 0.45 | 4 | 0.47 | 5 | 0.43 |
|  | CGC(R) | 1 | 0.05 | 0 | 0 | 1 | 0.09 |
|  | CGA(R) | 39 | 1.95 | 33 | 3.88 | 6 | 0.52 |
|  | CGG(R) | 6 | 0.3 | 0 | 0 | 6 | 0.52 |
| Ser^AGN^ (S) | AGU(S) | 54 | 1.18 | 22 | 0.8 | 32 | 1.75 |
|  | AGC(S) | 16 | 0.35 | 7 | 0.25 | 9 | 0.49 |
|  | AGA(S) | 54 | 2.7 | 14 | 1.65 | 40 | 3.48 |
|  | AGG(S) | 11 | 0.55 | 0 | 0 | 11 | 0.96 |
| Gly (G) | GGU(G) | 28 | 0.51 | 15 | 0.45 | 13 | 0.61 |
|  | GGC(G) | 25 | 0.46 | 11 | 0.33 | 14 | 0.66 |
|  | GGA(G) | 92 | 1.68 | 77 | 2.3 | 15 | 0.71 |
|  | GGG(G) | 74 | 1.35 | 31 | 0.93 | 43 | 2.02 |

Codon usage of the *Paradelphomyia curva* mt genome.

| Amino acid | Codon | N | RSCU | N+ | RSCU | N- | RSCU |
| --- | --- | --- | --- | --- | --- | --- | --- |
| Phe(F) | UUU(F) | 330 | 1.91 | 188 | 1.89 | 142 | 1.95 |
|  | UUC(F) | 15 | 0.09 | 11 | 0.11 | 4 | 0.05 |
| Leu^UUR^ (L) | UUA(L) | 512 | 5.22 | 269 | 5.06 | 243 | 5.42 |
|  | UUG(L) | 12 | 0.12 | 1 | 0.02 | 11 | 0.25 |
| Leu^CUN^ (L) | CUU(L) | 21 | 0.21 | 18 | 0.34 | 3 | 0.07 |
|  | CUC(L) | 4 | 0.04 | 4 | 0.08 | 0 | 0 |
|  | CUA(L) | 38 | 0.39 | 26 | 0.49 | 12 | 0.27 |
|  | CUG(L) | 1 | 0.01 | 1 | 0.02 | 0 | 0 |
| Ile (I) | AUU(I) | 386 | 1.85 | 250 | 1.86 | 136 | 1.83 |
|  | AUC(I) | 15 | 0.07 | 14 | 0.1 | 1 | 0.01 |
| Met (M) | AUA(M) | 225 | 1.08 | 139 | 1.03 | 86 | 1.16 |
|  | AUG(M) | 30 | 1 | 15 | 1 | 15 | 1 |
| Val (V) | GUU(V) | 76 | 1.84 | 37 | 1.51 | 39 | 2.33 |
|  | GUC(V) | 5 | 0.12 | 5 | 0.2 | 0 | 0 |
|  | GUA(V) | 76 | 1.84 | 55 | 2.24 | 21 | 1.25 |
|  | GUG(V) | 8 | 0.19 | 1 | 0.04 | 7 | 0.42 |
| Ser^UCN^ (S) | UCU(S) | 112 | 2.71 | 69 | 2.86 | 43 | 2.5 |
|  | UCC(S) | 9 | 0.22 | 8 | 0.33 | 1 | 0.06 |
|  | UCA(S) | 78 | 1.89 | 48 | 1.99 | 30 | 1.75 |
|  | UCG(S) | 3 | 0.07 | 2 | 0.08 | 1 | 0.06 |
| Pro (P) | CCU(P) | 70 | 2.07 | 50 | 2.02 | 20 | 2.22 |
|  | CCC(P) | 20 | 0.59 | 18 | 0.73 | 2 | 0.22 |
|  | CCA(P) | 40 | 1.19 | 28 | 1.13 | 12 | 1.33 |
|  | CCG(P) | 5 | 0.15 | 3 | 0.12 | 2 | 0.22 |
| Thr (T) | ACU(T) | 93 | 2.07 | 71 | 1.99 | 22 | 2.38 |
|  | ACC(T) | 17 | 0.38 | 15 | 0.42 | 2 | 0.22 |
|  | ACA(T) | 67 | 1.49 | 55 | 1.54 | 12 | 1.3 |
|  | ACG(T) | 3 | 0.07 | 2 | 0.06 | 1 | 0.11 |
| Ala (A) | GCU(A) | 86 | 2.07 | 48 | 1.79 | 38 | 2.58 |
|  | GCC(A) | 17 | 0.41 | 15 | 0.56 | 2 | 0.14 |
|  | GCA(A) | 51 | 1.23 | 36 | 1.35 | 15 | 1.02 |
|  | GCG(A) | 12 | 0.29 | 8 | 0.3 | 4 | 0.27 |
| Tyr (Y) | UAU(Y) | 161 | 1.85 | 79 | 1.76 | 82 | 1.95 |
|  | UAC(Y) | 13 | 0.15 | 11 | 0.24 | 2 | 0.05 |
| Stop (*) | UAA(*) | 0 | 0 | 0 | 0 | 0 | 0 |
|  | UAG(*) | 0 | 0 | 0 | 0 | 0 | 0 |
| His (H) | CAU(H) | 66 | 1.71 | 54 | 1.69 | 12 | 1.85 |
|  | CAC(H) | 11 | 0.29 | 10 | 0.31 | 1 | 0.15 |
| Gln (Q) | CAA(Q) | 69 | 1.89 | 49 | 1.88 | 20 | 1.9 |
|  | CAG(Q) | 4 | 0.11 | 3 | 0.12 | 1 | 0.1 |
| Asn (N) | AAU(N) | 183 | 1.84 | 118 | 1.79 | 65 | 1.94 |
|  | AAC(N) | 16 | 0.16 | 14 | 0.21 | 2 | 0.06 |
| Lys (K) | AAA(K) | 91 | 1.84 | 55 | 1.93 | 36 | 1.71 |
|  | AAG(K) | 8 | 0.16 | 2 | 0.07 | 6 | 0.29 |
| Asp (D) | GAU(D) | 58 | 1.76 | 39 | 1.7 | 19 | 1.9 |
|  | GAC(D) | 8 | 0.24 | 7 | 0.3 | 1 | 0.1 |
| Glu (E) | GAA(E) | 66 | 1.69 | 41 | 1.78 | 25 | 1.56 |
|  | GAG(E) | 12 | 0.31 | 5 | 0.22 | 7 | 0.44 |
| Cys (C) | UGU(C) | 38 | 1.9 | 11 | 1.83 | 27 | 1.93 |
|  | UGC(C) | 2 | 0.1 | 1 | 0.17 | 1 | 0.07 |
| Trp (W) | UGA(W) | 88 | 3 | 65 | 3 | 23 | 3 |
|  | UGG(W) | 12 | 1 | 3 | 1 | 9 | 1 |
| Arg (R) | CGU(R) | 12 | 0.58 | 3 | 0.26 | 9 | 0.98 |
|  | CGC(R) | 3 | 0.14 | 2 | 0.17 | 1 | 0.11 |
|  | CGA(R) | 35 | 1.68 | 28 | 2.4 | 7 | 0.76 |
|  | CGG(R) | 5 | 0.24 | 2 | 0.17 | 3 | 0.33 |
| Ser^AGN^ (S) | AGU(S) | 41 | 0.99 | 14 | 0.58 | 27 | 1.57 |
|  | AGC(S) | 5 | 0.12 | 4 | 0.17 | 1 | 0.06 |
|  | AGA(S) | 70 | 3.36 | 35 | 3 | 35 | 3.82 |
|  | AGG(S) | 0 | 0 | 0 | 0 | 0 | 0 |
| Gly (G) | GGU(G) | 50 | 0.94 | 22 | 0.7 | 28 | 1.29 |
|  | GGC(G) | 14 | 0.26 | 11 | 0.35 | 3 | 0.14 |
|  | GGA(G) | 80 | 1.5 | 60 | 1.9 | 20 | 0.92 |
|  | GGG(G) | 69 | 1.3 | 33 | 1.05 | 36 | 1.66 |

Codon usage of the *Rhipidia chenwenyoungi* mt genome.

| Amino acid | Codon | N | | RSCU | | N+ | | RSCU | | N- | | RSCU | |
| --- | --- | --- | --- | --- | --- | --- | --- | --- | --- | --- | --- | --- | --- |
| Phe(F) | UUU(F) | 310 | | 1.85 | | 176 | | 1.77 | | 134 | | 1.97 | |
|  | UUC(F) | 25 | | 0.15 | | 23 | | 0.23 | | 2 | | 0.03 | |
| Leu^UUR^ (L) | UUA(L) | 471 | | 5.27 | | 244 | | 5.08 | | 227 | | 5.49 | |
|  | UUG(L) | 16 | | 0.18 | | 5 | | 0.1 | | 11 | | 0.27 | |
| Leu^CUN^ (L) | CUU(L) | 19 | | 0.21 | | 17 | | 0.35 | | 2 | | 0.05 | |
|  | CUC(L) | 3 | | 0.03 | | 2 | | 0.04 | | 1 | | 0.02 | |
|  | CUA(L) | 26 | | 0.29 | | 19 | | 0.4 | | 7 | | 0.17 | |
|  | CUG(L) | 1 | | 0.01 | | 1 | | 0.02 | | 0 | | 0 | |
| Ile (I) | AUU(I) | 349 | | 1.79 | | 215 | | 1.88 | | 134 | | 1.67 | |
|  | AUC(I) | 17 | | 0.09 | | 17 | | 0.15 | | 0 | | 0 | |
| Met (M) | AUA(M) | 218 | | 1.12 | | 111 | | 0.97 | | 107 | | 1.33 | |
|  | AUG(M) | 17 | | 1 | | 5 | | 1 | | 12 | | 1 | |
| Val (V) | GUU(V) | 86 | | 2.18 | | 46 | | 1.96 | | 40 | | 2.5 | |
|  | GUC(V) | 8 | | 0.2 | | 7 | | 0.3 | | 1 | | 0.06 | |
|  | GUA(V) | 57 | | 1.44 | | 37 | | 1.57 | | 20 | | 1.25 | |
|  | GUG(V) | 7 | | 0.18 | | 4 | | 0.17 | | 3 | | 0.19 | |
| Ser^UCN^ (S) | UCU(S) | 98 | | 2.79 | | 53 | | 2.61 | | 45 | | 3.03 | |
|  | UCC(S) | 12 | | 0.34 | | 7 | | 0.34 | | 5 | | 0.34 | |
|  | UCA(S) | 88 | | 2.5 | | 58 | | 2.85 | | 30 | | 2.02 | |
|  | UCG(S) | 3 | | 0.09 | | 2 | | 0.1 | | 1 | | 0.07 | |
| Pro (P) | CCU(P) | 70 | | 2.24 | | 49 | | 2.18 | | 21 | | 2.4 | |
|  | CCC(P) | 6 | | 0.19 | | 6 | | 0.27 | | 0 | | 0 | |
|  | CCA(P) | 49 | | 1.57 | | 35 | | 1.56 | | 14 | | 1.6 | |
|  | CCG(P) | 0 | | 0 | | 0 | | 0 | | 0 | | 0 | |
| Thr (T) | ACU(T) | 88 | | 1.97 | | 63 | | 1.88 | | 25 | | 2.22 | |
|  | ACC(T) | 6 | | 0.13 | | 3 | | 0.09 | | 3 | | 0.27 | |
|  | ACA(T) | 83 | | 1.85 | | 67 | | 2 | | 16 | | 1.42 | |
|  | ACG(T) | 2 | | 0.04 | | 1 | | 0.03 | | 1 | | 0.09 | |
| Ala (A) | GCU(A) | 107 | | 2.61 | | 66 | | 2.54 | | 41 | | 2.73 | |
|  | GCC(A) | 14 | | 0.34 | | 10 | | 0.38 | | 4 | | 0.27 | |
|  | GCA(A) | 42 | | 1.02 | | 28 | | 1.08 | | 14 | | 0.93 | |
|  | GCG(A) | 1 | | 0.02 | | 0 | | 0 | | 1 | | 0.07 | |
| Tyr (Y) | UAU(Y) | 142 | | 1.72 | | 63 | | 1.54 | | 79 | | 1.9 | |
|  | UAC(Y) | 23 | | 0.28 | | 19 | | 0.46 | | 4 | | 0.1 | |
| Stop (*) | UAA(*) | 1 | | 0.03 | | 1 | | 0.05 | | 0 | | 0 | |
|  | UAG(*) | 0 | | 0 | | 0 | | 0 | | 0 | | 0 | |
| His (H) | CAU(H) | 65 | | 1.73 | | 50 | | 1.69 | | 15 | | 1.88 | |
|  | CAC(H) | 10 | | 0.27 | | 9 | | 0.31 | | 1 | | 0.13 | |
| Gln (Q) | CAA(Q) | 62 | | 1.85 | | 44 | | 1.96 | | 18 | | 1.64 | |
|  | CAG(Q) | 5 | | 0.15 | | 1 | | 0.04 | | 4 | | 0.36 | |
| Asn (N) | AAU(N) | 175 | 1.83 | | 106 | | 1.75 | | 69 | | 1.97 | |  |
|  | AAC(N) | 16 | | 0.17 | | 15 | | 0.25 | | 1 | | 0.03 | |
| Lys (K) | AAA(K) | 87 | | 2 | | 41 | | 2 | | 46 | | 2 | |
|  | AAG(K) | 0 | | 0 | | 0 | | 0 | | 0 | | 0 | |
| Asp (D) | GAU(D) | 56 | | 1.75 | | 37 | | 1.64 | | 19 | | 2 | |
|  | GAC(D) | 8 | | 0.25 | | 8 | | 0.36 | | 0 | | 0 | |
| Glu (E) | GAA(E) | 72 | | 1.92 | | 40 | | 2 | | 32 | | 1.83 | |
|  | GAG(E) | 3 | | 0.08 | | 0 | | 0 | | 3 | | 0.17 | |
| Cys (C) | UGU(C) | 36 | | 1.89 | | 12 | | 1.71 | | 24 | | 2 | |
|  | UGC(C) | 2 | | 0.11 | | 2 | | 0.29 | | 0 | | 0 | |
| Trp (W) | UGA(W) | 89 | | 2.97 | | 61 | | 2.95 | | 28 | | 3 | |
|  | UGG(W) | 2 | | 1 | | 0 | | 0 | | 2 | | 1 | |
| Arg (R) | CGU(R) | 20 | | 0.82 | | 9 | | 0.75 | | 11 | | 0.88 | |
|  | CGC(R) | 1 | | 0.04 | | 1 | | 0.08 | | 0 | | 0 | |
|  | CGA(R) | 27 | | 1.1 | | 22 | | 1.83 | | 5 | | 0.4 | |
|  | CGG(R) | 5 | | 0.2 | | 3 | | 0.25 | | 2 | | 0.16 | |
| Ser^AGN^ (S) | AGU(S) | 9 | | 0.26 | | 1 | | 0.05 | | 8 | | 0.54 | |
|  | AGC(S) | 1 | | 0.03 | | 1 | | 0.05 | | 0 | | 0 | |
|  | AGA(S) | 82 | | 3.35 | | 36 | | 3 | | 46 | | 3.68 | |
|  | AGG(S) | 12 | | 0.49 | | 1 | | 0.08 | | 11 | | 0.88 | |
| Gly (G) | GGU(G) | 45 | | 0.87 | | 25 | | 0.84 | | 20 | | 0.92 | |
|  | GGC(G) | 7 | | 0.14 | | 5 | | 0.17 | | 2 | | 0.09 | |
|  | GGA(G) | 100 | | 1.94 | | 66 | | 2.22 | | 34 | | 1.56 | |
|  | GGG(G) | 54 | | 1.05 | | 23 | | 0.77 | | 31 | | 1.43 | |

Codon usage of the *Cylindrotoma* sp. mt genome.

| Amino acid | Codon | N | RSCU | N+ | RSCU | N- | RSCU |
| --- | --- | --- | --- | --- | --- | --- | --- |
| Phe(F) | UUU(F) | 299 | 1.75 | 154 | 1.59 | 145 | 1.96 |
|  | UUC(F) | 43 | 0.25 | 40 | 0.41 | 3 | 0.04 |
| Leu^UUR^ (L) | UUA(L) | 511 | 4.99 | 270 | 4.78 | 241 | 5.24 |
|  | UUG(L) | 28 | 0.27 | 3 | 0.05 | 25 | 0.54 |
| Leu^CUN^ (L) | CUU(L) | 33 | 0.32 | 29 | 0.51 | 4 | 0.09 |
|  | CUC(L) | 0 | 0 | 0 | 0 | 0 | 0 |
|  | CUA(L) | 41 | 0.4 | 35 | 0.62 | 6 | 0.13 |
|  | CUG(L) | 2 | 0.02 | 2 | 0.04 | 0 | 0 |
| Ile (I) | AUU(I) | 325 | 1.77 | 212 | 1.77 | 113 | 1.77 |
|  | AUC(I) | 26 | 0.14 | 25 | 0.21 | 1 | 0.02 |
| Met (M) | AUA(M) | 201 | 1.09 | 123 | 1.02 | 78 | 1.22 |
|  | AUG(M) | 24 | 1 | 13 | 1 | 11 | 1 |
| Val (V) | GUU(V) | 86 | 1.88 | 37 | 1.35 | 49 | 2.68 |
|  | GUC(V) | 6 | 0.13 | 6 | 0.22 | 0 | 0 |
|  | GUA(V) | 86 | 1.88 | 65 | 2.36 | 21 | 1.15 |
|  | GUG(V) | 5 | 0.11 | 2 | 0.07 | 3 | 0.16 |
| Ser^UCN^ (S) | UCU(S) | 120 | 2.66 | 52 | 1.97 | 68 | 3.61 |
|  | UCC(S) | 12 | 0.27 | 11 | 0.42 | 1 | 0.05 |
|  | UCA(S) | 79 | 1.75 | 67 | 2.54 | 12 | 0.64 |
|  | UCG(S) | 6 | 0.13 | 5 | 0.19 | 1 | 0.05 |
| Pro (P) | CCU(P) | 77 | 2.35 | 50 | 2.06 | 27 | 3.18 |
|  | CCC(P) | 15 | 0.46 | 12 | 0.49 | 3 | 0.35 |
|  | CCA(P) | 37 | 1.13 | 34 | 1.4 | 3 | 0.35 |
|  | CCG(P) | 2 | 0.06 | 1 | 0.04 | 1 | 0.12 |
| Thr (T) | ACU(T) | 106 | 2.04 | 71 | 1.76 | 35 | 2.98 |
|  | ACC(T) | 21 | 0.4 | 19 | 0.47 | 2 | 0.17 |
|  | ACA(T) | 80 | 1.54 | 71 | 1.76 | 9 | 0.77 |
|  | ACG(T) | 1 | 0.02 | 0 | 0 | 1 | 0.09 |
| Ala (A) | GCU(A) | 113 | 2.43 | 61 | 2.02 | 52 | 3.2 |
|  | GCC(A) | 25 | 0.54 | 23 | 0.76 | 2 | 0.12 |
|  | GCA(A) | 46 | 0.99 | 36 | 1.19 | 10 | 0.62 |
|  | GCG(A) | 2 | 0.04 | 1 | 0.03 | 1 | 0.06 |
| Tyr (Y) | UAU(Y) | 125 | 1.55 | 57 | 1.34 | 68 | 1.79 |
|  | UAC(Y) | 36 | 0.45 | 28 | 0.66 | 8 | 0.21 |
| Stop (*) | UAA(*) | 0 | 0 | 0 | 0 | 0 | 0 |
|  | UAG(*) | 0 | 0 | 0 | 0 | 0 | 0 |
| His (H) | CAU(H) | 56 | 1.49 | 43 | 1.39 | 13 | 2 |
|  | CAC(H) | 19 | 0.51 | 19 | 0.61 | 0 | 0 |
| Gln (Q) | CAA(Q) | 68 | 1.79 | 52 | 1.96 | 16 | 1.39 |
|  | CAG(Q) | 8 | 0.21 | 1 | 0.04 | 7 | 0.61 |
| Asn (N) | AAU(N) | 178 | 1.75 | 106 | 1.64 | 72 | 1.92 |
|  | AAC(N) | 26 | 0.25 | 23 | 0.36 | 3 | 0.08 |
| Lys (K) | AAA(K) | 76 | 1.73 | 40 | 1.82 | 36 | 1.64 |
|  | AAG(K) | 12 | 0.27 | 4 | 0.18 | 8 | 0.36 |
| Asp (D) | GAU(D) | 49 | 1.48 | 30 | 1.3 | 19 | 1.9 |
|  | GAC(D) | 17 | 0.52 | 16 | 0.7 | 1 | 0.1 |
| Glu (E) | GAA(E) | 77 | 1.97 | 44 | 1.96 | 33 | 2 |
|  | GAG(E) | 1 | 0.03 | 1 | 0.04 | 0 | 0 |
| Cys (C) | UGU(C) | 35 | 1.71 | 8 | 1.33 | 27 | 1.86 |
|  | UGC(C) | 6 | 0.29 | 4 | 0.67 | 2 | 0.14 |
| Trp (W) | UGA(W) | 96 | 3 | 71 | 3 | 25 | 3 |
|  | UGG(W) | 5 | 1 | 0 | 0 | 5 | 1 |
| Arg (R) | CGU(R) | 14 | 0.75 | 4 | 0.4 | 10 | 1.15 |
|  | CGC(R) | 2 | 0.11 | 2 | 0.2 | 0 | 0 |
|  | CGA(R) | 32 | 1.71 | 27 | 2.7 | 5 | 0.58 |
|  | CGG(R) | 9 | 0.48 | 4 | 0.4 | 5 | 0.58 |
| Ser^AGN^ (S) | AGU(S) | 44 | 0.97 | 16 | 0.61 | 28 | 1.49 |
|  | AGC(S) | 10 | 0.22 | 7 | 0.27 | 3 | 0.16 |
|  | AGA(S) | 55 | 2.95 | 23 | 2.3 | 32 | 3.69 |
|  | AGG(S) | 0 | 0 | 0 | 0 | 0 | 0 |
| Gly (G) | GGU(G) | 38 | 0.71 | 20 | 0.62 | 18 | 0.85 |
|  | GGC(G) | 2 | 0.04 | 2 | 0.06 | 0 | 0 |
|  | GGA(G) | 108 | 2.02 | 84 | 2.6 | 24 | 1.13 |
|  | GGG(G) | 66 | 1.23 | 23 | 0.71 | 43 | 2.02 |
